# Mitochondrial Homeostasis–Related lncRNAs are Potential Biomarkers for Predicting Prognosis and Immune Response in Lung Adenocarcinoma

**DOI:** 10.3389/fgene.2022.870302

**Published:** 2022-06-13

**Authors:** Bo Peng, Han Lou, Chen Chen, Lei Wang, Huawei Li, Tong Lu, Ruisi Na, Ran Xu, Tong Xin, Lingqi Yao, Henghui Xu, Kaiyu Wang, Xin Liu, Linyou Zhang

**Affiliations:** ^1^ Department of Thoracic Surgery, The Second Affiliated Hospital of Harbin Medical University, Harbin, China; ^2^ Department of Pharmacology (State-Province Key Laboratories of Biomedicine-Pharmaceutics of China and Key Laboratory of Cardiovascular Medicine Research, Ministry of Education), College of Pharmacy, Harbin Medical University, Harbin, China; ^3^ Department of Gastrointestinal Medical Oncology, Harbin Medical University Cancer Hospital, Harbin, China; ^4^ The Fourth Department of Medical Oncology, Harbin Medical University Cancer Hospital, Harbin, China

**Keywords:** mitochondrial homeostasis, long noncoding RNAs, lung adenocarcinoma, nomogram, prognosis, immunotherapy

## Abstract

The prognosis of the most common histological subtype of lung cancer, lung adenocarcinoma (LUAD), is relatively poor. Mitochondrial homeostasis depends to a great extent on the coordination between mitophagy and mitochondrial biogenesis, the deregulation of which causes various human diseases, including cancer. There is accumulating evidence that long noncoding RNAs (lncRNAs) are critical in predicting the prognosis and immune response in carcinoma. Therefore, it is critical to discern lncRNAs related to mitochondrial homeostasis in LUAD patients. In this study, we identified mitochondrial homeostasis–related lncRNAs (MHRlncRNAs) by coexpression analysis. In order to construct a prognostic signature composed of three MHRlncRNAs, univariate and multivariate Cox regression analyses were performed. Kaplan–Meier analysis, stratification analysis, principal component analysis (PCA), receiver operating characteristic (ROC) curve, gene set enrichment analysis (GSEA), and nomogram were applied to evaluate and optimize the risk model. Subsequently, we identified the mitochondrial homeostasis–related lncRNA signature (MHLncSig) as an independent predictive factor of prognosis. Based on the LUAD subtypes regrouped by this risk model, we further investigated the underlying tumor microenvironment, tumor mutation burden, and immune landscape behind different risk groups. Likewise, individualized immunotherapeutic strategies and candidate compounds were screened to aim at different risk subtypes of LUAD patients. Finally, we validated the expression trends of lncRNAs included in the risk model using quantitative real-time polymerase chain reaction (qRT-PCR) assays. The established MHLncSig may be a promising tool for predicting the prognosis and guiding individualized treatment in LUAD.

## Introduction

As the most commonly diagnosed pathological subtype of lung cancer, the incidence of lung adenocarcinoma (LUAD) is increasing globally every year. LUAD patients have poor prognosis which commonly results from late detection and individual treatment differences. Compared with the conventional treatments including surgery and chemotherapy, the development of multiple agents targeting driver gene mutations showed appreciable promise in LUAD treatment due to the advances of cancer genomics (Denisenko et al., 2018; Joseph et al., 2018). Unfortunately, secondary mutations in tumors usually contribute to targeted therapeutic resistance (Liu et al., 2018). In recent years, advance of cancer immunology makes immunotherapy a hotspot in the clinical treatment of LUAD. Classical immune checkpoint inhibitors (ICIs) including antiprogrammed cell death 1 (PD-1) and antiprogrammed cell death-ligand 1 (PD-L1) agents exert a persisting and powerful antitumor effect in LUAD patients (Forde et al., 2018). However, only a proportion of patients could benefit from immunotherapy because of the relatively low overall response rate of ICI (Li et al., 2018a). For the aforementioned reasons, the 5-year survival rate of LUAD remains inadequate (Wang et al., 2021; Zhu et al., 2022). Therefore, novel molecular biomarkers are urgently required to predict the prognosis and therapeutic response for LUAD patients.

Mitochondria are essential organelles that regulate ATP production and energy transformation *via* oxidative phosphorylation (OXPHOS) and the tricarboxylic acid cycle (TCA), which also modulate iron metabolism, Ca^2+^ signaling, innate immunity, and apoptotic cell death in mammalian cells (Zong et al., 2016; Pathak and Trebak, 2018). Mitochondria, generally defined as highly motile and plastic organelles, constantly undergo processes of fusion and fission and update through mitophagy and mitochondrial biogenesis to maintain homeostasis (Ma et al., 2020). In most cases, mitochondrial DNA (mtDNA) mutations, deleted, or damaged DNA replication induce the dysbiosis of mitochondrial homeostasis and consequent mitochondrial dysfunction (Butow and Avadhani, 2004; Wallace, 2012). Mitochondrial dysfunction caused by significant abnormalities in the mtDNA copy number is intimately associated with many diseases such as age-related pathologies, mtDNA depletion syndrome, and several carcinomas (Park et al., 2009; Imanishi et al., 2011; Greaves et al., 2012). The results from previous studies indicated that low copy numbers of mtDNA are widely observed in multiple cancers including colon, breast, hepatocellular carcinomas, prostate cancer, and astrocytoma (Horton et al., 1996; Lee et al., 2005; Petros et al., 2005; Tseng et al., 2006; Correia et al., 2011). Moreover, mtDNA depletion induced by experimental methods promotes aggressive phenotype in prostate and colorectal cancer cells (Moro et al., 2009; Guo et al., 2011).

Long noncoding RNAs (lncRNAs), a subclass of noncoding RNAs with a length of >200 nucleotides, have been confirmed to be involved in the tumorigenesis and progression of various tumors, including LUAD (Zheng et al., 2021). Recent studies have revealed that dysregulation of specific lncRNAs was inextricably associated with tumor proliferation, metastasis, and drug resistance in lung cancer (Mao et al., 2018; Wang et al., 2019a; Yang et al., 2020). Nonetheless, the specific function of lncRNAs in mitochondrial homeostasis remains to be clarified. Therefore, investigating the potential mechanism of mitochondrial homeostasis–related lncRNAs (MHRlncRNAs) in LUAD may be valuable for prognostic biomarkers.

In our study, we extracted the expression profiles of 1,499 mitochondrial homeostasis-related genes (MHRGs) from the publicly available dataset: The Cancer Genome Atlas (TCGA) dataset. Using Pearson’s correlation analysis, we identified 2,850 lncRNAs coexpressed with MHRGs. Next, mitochondrial homeostasis–related lncRNA signature (MHLncSig) was constructed to forecast the survival of LUAD patients utilizing differential expression analysis, univariate and multivariate Cox regression analyses. This personalized and robust prognostic signature is not only an independent indicator of overall survival, but also significantly related to tumor microenvironment, tumor mutation burden (TMB), immune infiltration, immunotherapeutic efficacy, and drug sensitivity.

## Materials and Methods

### Data Acquisition and Study Design

**FIGURE 1:**
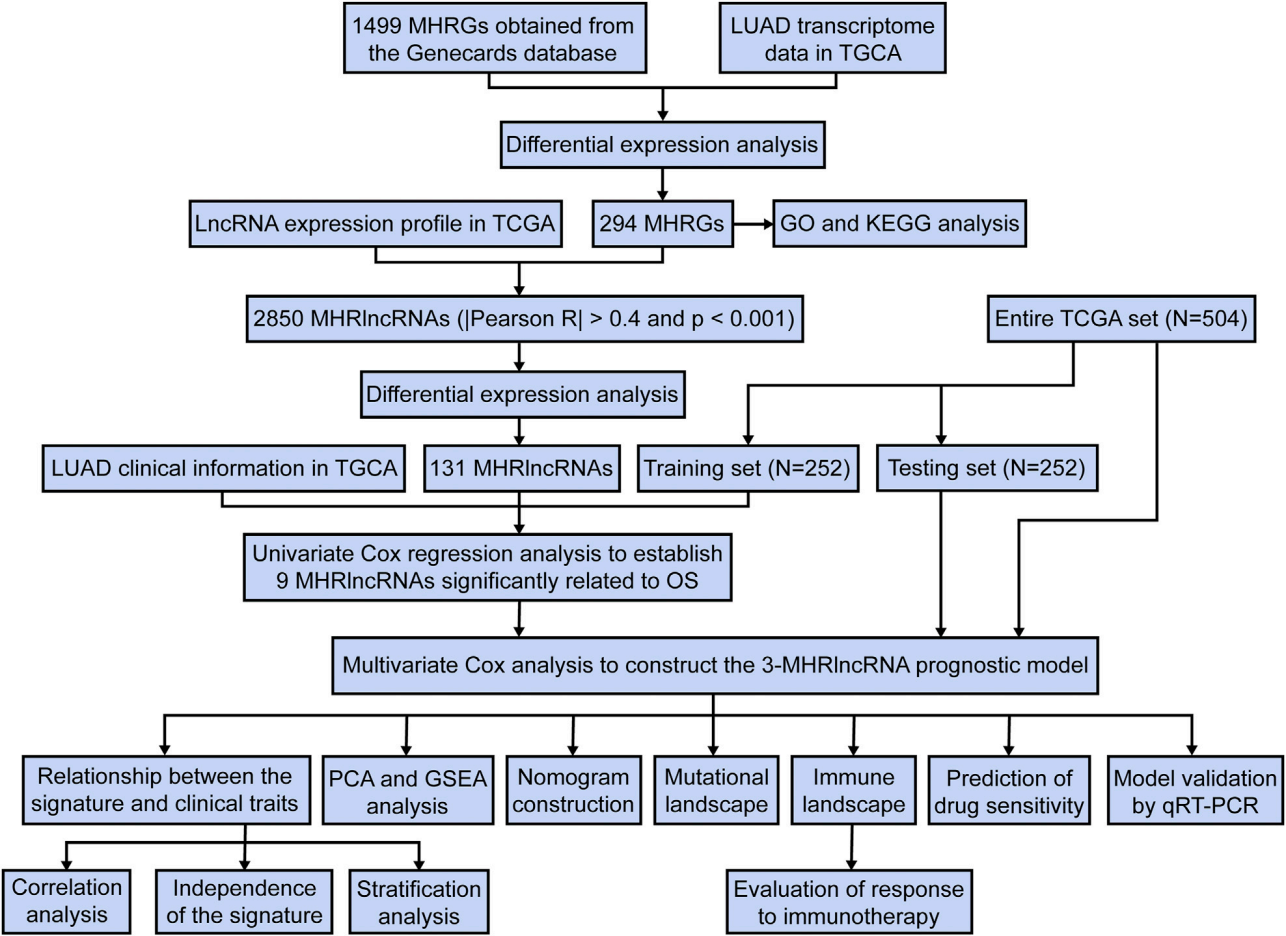
A concise flow diagram of this study.

We downloaded the RNA transcriptome sequence data, corresponding clinical features, and mutation data for the patients with LUAD from The Cancer Genome Atlas (TCGA) (https://cancergenome.nih.gov/) database. The patients with missing survival information were removed from this study. The study flowchart is presented in Figure 1.

### Selection of Mitochondrial Homeostasis-Related Genes and lncRNAs

1,499 MHRGs with the relevance score >4 were obtained from the GeneCards (https://www.genecards.org/) database, including mitochondrial DNA-encoded genes and genomic DNA-encoded genes that regulate mitochondrial homeostasis *via* multiple pathways. We extracted the expression profile of MHRGs from the downloaded transcriptome data and performed differential expression analysis between the normal samples and LUAD samples. 294 differentially expressed MHRGs (|Log fold change (FC)| > 1 and false discovery rate (FDR) adjusted *p* < 0.05) were considered to be closely related to the pathogenesis and progression of LUAD. Next, we used Pearson’s correlation analysis and identified 2,850 MHRlncRNAs (|Pearson R| > 0.4 and *p* < 0.001). Further differential expression analysis based on 2,850 MHRlncRNAs was conducted to discern candidate lncRNAs for model building.

### Establishment of the Risk Signature

We identified 131 differentially expressed MHRlncRNAs as candidate lncRNAs from the 2,850 MHRlncRNAs (|log FC| > 1 and FDR <0.05). The TCGA set was randomly separated into training (252 patients) and testing sets (252 patients), and no significant differences in the clinical properties was observed. Table 1 summarized the clinical features of these three sets. We used the training set to construct the prognostic signature The testing set and the entire TCGA set were utilized to validate the established signature. Univariate Cox regression analysis was performed to screen nine lncRNAs with significant prognostic value from 131 differentially expressed MHRlncRNAs. Multivariate Cox regression analysis was utilized to analyze and further confirm the prognostic significance from the previous steps, and three lncRNAs were retained during multiple computing. Thereafter, according to a linear combination of expression levels weighted with the regression coefficients calculated by multivariate Cox regression analysis, we established a risk formula that can calculate the risk score for each patient with LUAD

$$MHLncSig\left(risk\;score\right)=\sum_{i=1}^{n}coefficient\left(\text{lncRNAi}\right)*\;expression\;level\left(\text{lncRNAi}\right)\;\;,$$

where n indicates the amount of MHRlncRNAs included in the prognostic model, and the multivariate Cox regression analysis provided the coefficient for lncRNAi. According to the median risk score, we further distinguished the high- and low-risk subgroups of LUAD.

**TABLE 1:** Clinical features of the three LUAD patients sets.

Covariate		Training set (*n* = 252)	Testing set (*n* = 252)	TCGA set (*n* = 504)	*p*-value
Age, no (%)	≤65	117 (46.43)	121 (48.02)	238 (47.22)	0.721
	>65	131 (51.98)	125 (49.6)	256 (50.79)	
	unknown	4 (1.59)	6 (2.38)	10 (1.98)	
Gender, no (%)	female	129 (51.19)	141 (55.95)	270 (53.57)	0.326
	male	123 (48.81)	111 (44.05)	234 (46.43)	
T stage, no (%)	T1–2	217 (86.11)	220 (87.3)	437 (86.71)	0.701
	T3–4	34 (13.49)	30 (11.9)	64 (12.7)	
	unknown	1 (0.4)	2 (0.79)	3 (0.6)	
N stage, no (%)	N0	161 (63.89)	164 (65.08)	325 (64.48)	0.849
	N1–3	85 (33.73)	82 (32.54)	167 (33.13)	
	unknown	6 (2.38)	6 (2.38)	12 (2.38)	
M stage, no (%)	M0	169 (67.06)	166 (65.87)	335 (66.47)	1
	M1	13 (5.16)	12 (4.76)	25 (4.96)	
	unknown	70 (27.78)	74 (29.37)	144 (28.57)	
Pathologic stage, no (%)	I–II	191 (75.79)	198 (78.57)	389 (77.18)	0.629
	III–IV	56 (22.22)	51 (20.24)	107 (21.23)	
	unknown	5 (1.98)	3 (1.19)	8 (1.59)	

LUAD, lung adenocarcinoma.

### Function Enrichment Analysis

To discern the potential biological roles and metabolic pathways in differentially expressed MHRGs, the Gene Ontology (GO) categories and Kyoto Encyclopedia of Genes and Genomes (KEGG) pathways were identified in the R program. We also used the Gene Set Enrichment Analysis (GSEA) (version 4.1.0) to investigate the biological processes and metabolic pathways involved in the different risk subgroups of LUAD stratified by MHLncSig. In terms of the reference file, c2.cp.kegg.v7.4.symbols.gmt was used and FDR <0.05 was considered significant.

### Tumor Microenvironment and Tumor Mutation Burden (TMB)

R “estimate” package was utilized to calculate the stromal score, immune score, and ESTIMATE score for each LUAD patient, which represents the infiltration of both the stromal and immune cells in tumor tissues. We downloaded the somatic mutation data from the TCGA database and calculated the TMB score for each LUAD sample using a Perl script.

### Immune Landscape and Immunotherapeutic Response Based on the MHLncSig

In order to calculate the relative abundance of 22 kinds of tumor-infiltrating immune cells (TICs) for each sample in the TCGA cohort, we used the CIBERSORT method. Subsequently, the Wilcoxon test was employed to verify the differentiation of 22 types of TICs between the low- and high-risk groups. Next, we converted the gene expression profile of tumor samples into immune function–related score and performed differential analysis of immune function between different risk subgroups using R “limma,” “GSVA,” “GSEABase,” “ggpubr,” and “reshape2” packages. Finally, we performed the Tumor Immune Dysfunction and Exclusion (TIDE) algorithm to access the response to immunotherapy.

### Screening the Potential Compounds for Clinical Treatment of LUAD Risk Subgroups

We computed the IC50 of antitumor drugs that are commonly recommended for LUAD treatment based on gene expression profile of LUAD patients using the “pRRophetic” package in R software. The Wilcoxon test was adopted to evaluate the difference in the IC50 levels between distinct subgroups.

### RNA Isolation and qRT-PCR

Patient samples comprising a cohort of 15 paired LUAD and adjacent normal tissues were collected between October 2021 and December 2021 from the Second Affiliated Hospital of Harbin Medical University. With the approval for experiments from the Ethics and Scientific Committees of the Second Affiliated Hospital of Harbin Medical University (Approval Number: KY2021-375), written informed consent was provided by all enrolled patients. The total RNA was extracted using the TRIzol reagent (Invitrogen, CA, United States). 1 µg total RNA and ReverTra Ace qPCR RT Master Mix (TOYOBO) were used for reverse transcriptase reaction. Next, 1 µl synthesized cDNA was used in PCR amplification. The levels of three lncRNAs were measured quantitatively by the SYBR Green Master Mix Kit (TOYOBO). GAPDH was selected as an internal reference. The relative expression was calculated based on the comparative Ct (2^−ΔΔCt^) method, and Student’s t-test (two-tailed) was utilized to assess the significance of lncRNA expression differences in GraphPad Prism (version 8.0). The qRT-PCR primers in this study were as follows:

FENDRR
Forward: 5′-GCC​TCA​GAG​TGG​GCT​AGA​TT-3′
Reverse: 5′-TAA​CGA​TCC​CAC​CAA​CAC​CA-3′
AL590666.2
Forward: 5′-ACA​GAA​TGA​TCC​AGG​CAC​CA-3′
Reverse: 5′-AGG​ACA​AGA​TGG​ACG​CAG​AT-3′
AC090559.1
Forward: 5′-TGC​TAG​GCA​ATT​CTG​GAA​GC-3′
Reverse: 5′-TTG​CTG​TTG​CCA​CAA​AGT​GA-3′
GAPDH
Forward: 5′-CAT​GTT​CGT​CAT​GGG​TGT​GAA-3′
Reverse: 5′-GGC​ATG​GAC​TGT​GGT​CAT​GAG-3′

### Statistical Analyses

We performed all bioinformatics analyses in R-version 4.1.1. Survival curves were plotted using Kaplan–Meier analysis to estimate the difference of survival for both the subgroups of LUAD in the R “survival” and “survminer” packages. Receiver operating characteristic (ROC) curves and the area under the curve (AUC) values were applied to evaluate the degree of robustness and accuracy of our MHLncSig. Principal component analysis (PCA) was utilized to reduce the dimension of high-dimensional data and visualize the differentiation between the risk subgroups in terms of entire genes expression in TCGA, 2,850 MHRlncRNAs, and the risk model of the 3 MHRlncRNAs. The R “limma” and “scatterplot3d” packages were employed to enable this process. In addition, we constructed a nomogram for predicting the overall survival of 1, 3, and 5 years, and a correction curve was applied to assess the consistency between the model prediction outcome and practical outcome by using the R “survival,” “survminer,” “rms,” and “regplot” packages, respectively. We set the statistically significant threshold as *p*-value <0.05 (*p < 0.05, **p < 0.01, and ***p < 0.001).

## Results

### Identification of Mitochondrial Homeostasis–Related lncRNAs in LUAD Patients

**FIGURE 2:**
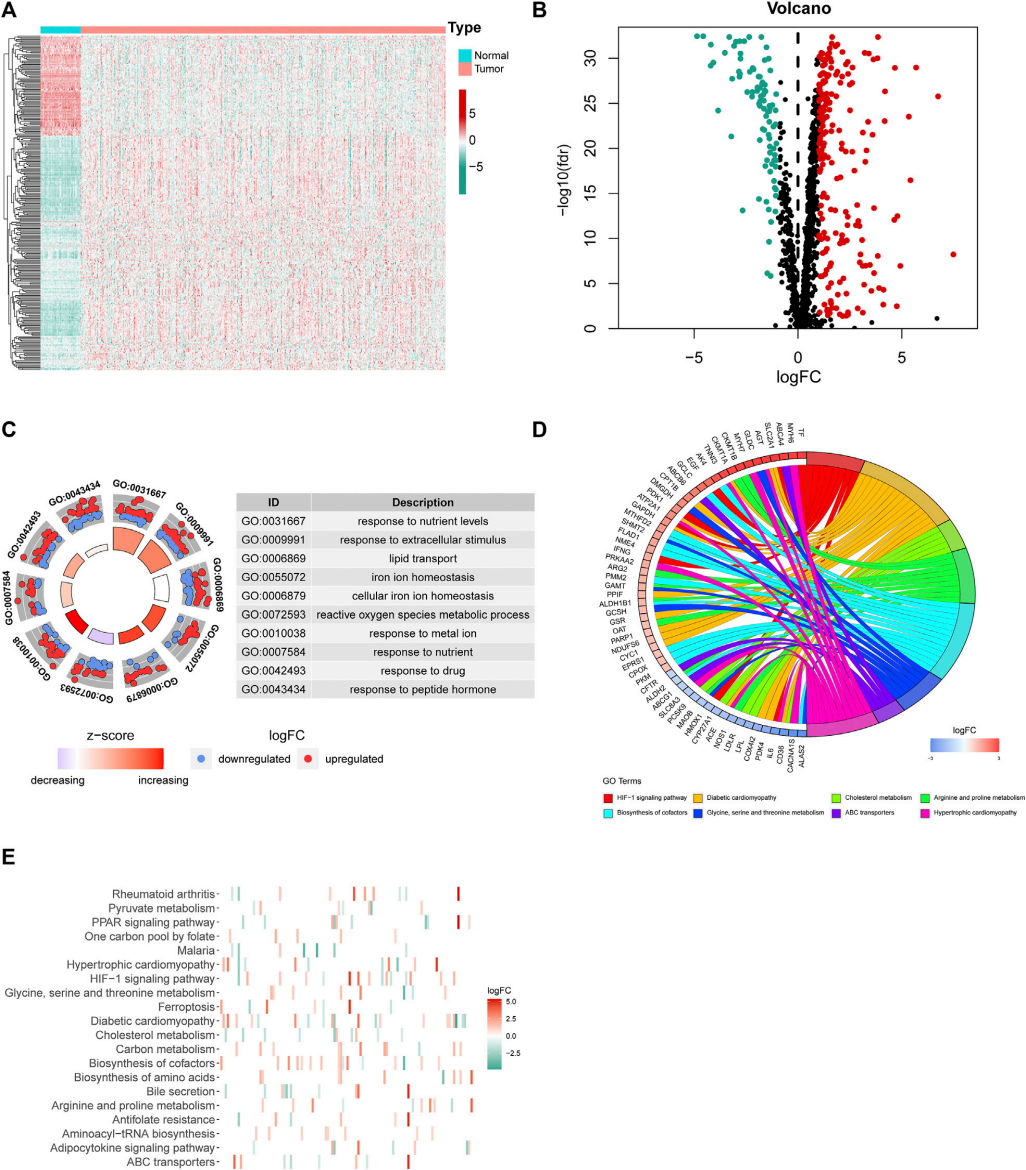
Differential expression analysis and corresponding functional enrichment of the 1,499 MHRGs. **(A)** A heatmap of the 294 differentially expressed genes. **(B)** A volcano plot based on the 205 upregulated and 89 downregulated genes. **(C)** The most highly significant 10 results in the GO analysis. **(D,E)** The corresponding genes and the distribution of enriched biological pathways in the KEGG analysis.

First, we downloaded the coding gene list associated with mitochondrial homeostasis from the GeneCards database. Based on the expression profile of LUAD and normal subjects in TCGA, differential expression analysis of 1,499 MHRGs was conducted to discern the genes that may serve a role in tumorigenesis. A heatmap and a volcano map were plotted to illustrate the 294 differentially expressed genes (205 upregulated genes and 89 downregulated genes in LUAD) (Figures 2A,B), and the detailed data are summarized in Supplementary Table S1. Furthermore, GO and KEGG analyses were utilized to explore the molecular functions and pathways behind these 294 genes. Figure 2C visualized the most highly significant cellular components, biological processes, and molecular functions. Moreover, biological pathways were mainly enriched in processes associated with mitochondrial synthesis and metabolism (Figures 2D,E).

**FIGURE 3:**
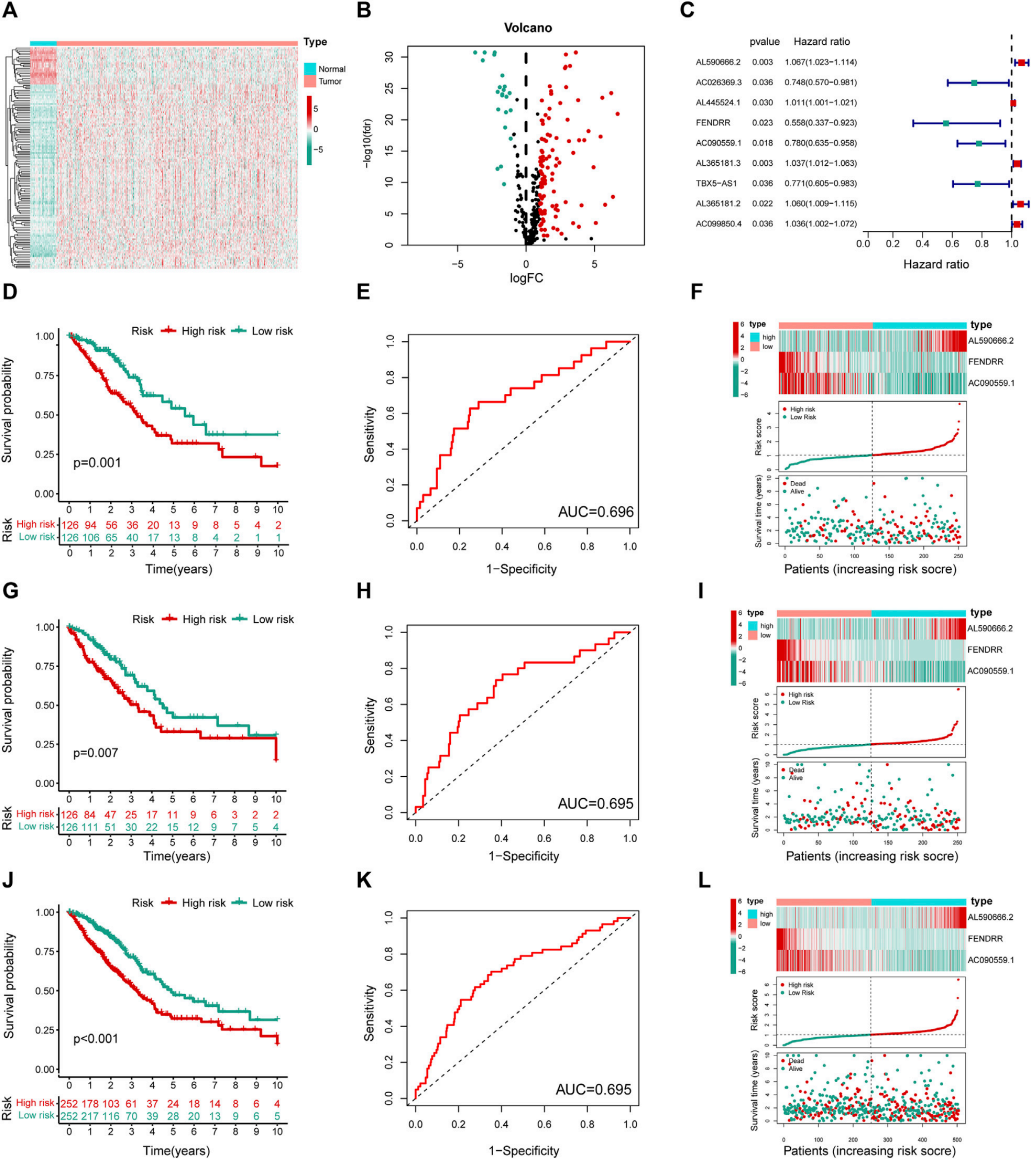
Construction and validation of the MHLncSig. **(A)** A heatmap of differential expression analysis based on 2,850 MHRlncRNAs. **(B)** A volcano plot based on the 109 upregulated MHRlncRNAs and 22 downregulated MHRlncRNAs. **(C)** A forest plot of nine MHRlncRNAs significantly related to survival in LUAD. **(D,G,J)** Different Kaplan–Meier curves in the two risk groups stratified by MHLncSig in the training set, testing set, and the entire TCGA set. **(E,H,K)** ROC curve of the MHLncSig at 3 years in the training set, testing set, and the entire TCGA set. **(F,I,L)** The expression patterns of MHLncSig, the risk grade distribution, and the survival status between the low- and high-risk groups in the training set, testing set, and the entire TCGA set.

Next, 2,850 lncRNAs coexpressed with the 294 differentially expressed genes were identified as MHRlncRNAs by Pearson’s correlation analysis. Furthermore, differential expression analysis based on the 2,850 lncRNAs was utilized to screen the candidate MHRlncRNAs for model construction. The result of differential analysis is summarized in Supplementary Table S2 and visualized in Figures 3A,B.

### Construction and Validation of the MHLncSig in LUAD Patients

To investigate the prognostic value of 131 candidate MHRlncRNAs, we performed univariate Cox analysis in the training set (252 patients) and identified nine MHRlncRNAs which are significantly associated with the survival of LUAD (Figure 3C). According to the multivariate Cox proportional hazards regression analysis, AL590666.2, FENDRR, and AC090559.1 were further established as components of the risk model. The details of multivariate Cox regression analysis are shown in Table 2. Finally, we designed a risk-score formula for LUAD patients’ survival prediction. The risk score formula is as follows: risk score = 0.063 × expression quantity of AL590666.2 + (−0.527) × expression quantity of FENDRR + (−0.158) × expression quantity of AC090559.1. AL590666.2 with a positive coefficient tended to be a detrimental factor. However, the other two lncRNAs including FENDRR and AC090559.1 tended to be protective factors. Next, grouping was conducted based on the median risk score of the training set; the Kaplan–Meier plot showed entirely different survival curves between different groups (*p* = 0.001, Figure 3D). The AUC value of the receiver operating characteristic (ROC) curve was 0.696, indicating that MHLncSig is equipped with quite accurate prediction performance for the prognosis (Figure 3E). In addition, the expression standards of MHLncSig for each patient, the risk grade distribution, and the survival status between the two groups are depicted in Figure 3F.

**TABLE 2:** Multivariate Cox regression analysis of three prognostic lncRNAs.

LncRNA	Coefficient	HR	HR.95L	HR.95H	*p*-value
AL590666.2	0.063	1.066	1.019	1.114	0.005
FENDRR	−0.527	0.590	0.348	1.002	0.051
AC090559.1	−0.158	0.854	0.691	1.055	0.144

HR, hazard ratio; CI, confidence interval.

Next, we validated our MHLncSig in the testing set (252 patients) and the entire TCGA set (504 patients). The survival analysis indicated that the patients in the low-risk group had better overall survival (OS) than that in the high-risk group (Figure 3G). The AUC value of MHLncSig in the testing set reached 0.695 (Figure 3H). Figure 3I shows the expression patterns of MHLncSig, the risk grade distribution, and the survival of LUAD patients. Notably, the entire TCGA set also manifested similar results as the aforementioned findings (Figures 3J–L).

### Relationship Between MHLncSig and Clinical Traits

We next performed correlation analysis between the risk score calculated by MHLncSig and clinical traits including age, gender, T stage, N stage, M stage, and pathological stage. The results indicated that there were high correlations between MHLncSig and all these clinical features in the entire TCGA set (*p* < 0.05, Supplementary Figure S1). Furthermore, we evaluated the predictive performance of our MHLncSig in different subgroups classified by clinical traits. In the vast majority of subgroups of LUAD patients, the low-risk group continued to maintain supremacy in OS (Supplementary Figure S2). Moreover, univariate and multivariate Cox regression analyses were performed to investigate whether the MHLncSig had independent prognostic effect for LUAD. After correction for other clinical variables, we observed that the MHLncSig retained independent significance for the prediction of OS in the training set, the testing set, and the entire TCGA set (Table 3).

**TABLE 3:** Univariate and multivariate Cox regression analysis of the MHLncSig and prognosis.

Variable	Univariate model			Multivariate model		
	HR	95% CI	*p*-value	HR	95% CI	*p*-value
Training set (*n* = 252)						
Age	1.030	1.005–1.055	0.016	1.031	1.007–1.056	0.012
Gender	0.957	0.628–1.460	0.840			
Stage	1.464	1.201–1.784	<0.001	1.289	1.044–1.592	0.018
Risk score	2.098	1.529–2.878	<0.001	1.804	1.265–2.573	0.001
Testing set (*n* = 252)						
Age	0.992	0.973–1.012	0.451			
Gender	1.295	0.856–1.960	0.221			
Stage	1.879	1.535–2.300	<0.001	1.869	1.526–2.289	<0.001
Risk score	1.369	1.127–1.662	0.002	1.376	1.116–1.698	0.003
TCGA set (*n* = 504)						
Age	1.008	0.993–1.023	0.309			
Gender	1.102	0.820–1.479	0.520			
Stage	1.629	1.417–1.872	<0.001	1.550	1.349–1.782	<0.001
Risk score	1.510	1.297–1.759	<0.001	1.415	1.188–1.684	<0.001

HR, hazard ratio; CI, confidence interval.

### Evaluation of the Prognostic MHLncSig in Terms of PCA and GSEA Analyses

**FIGURE 4:**
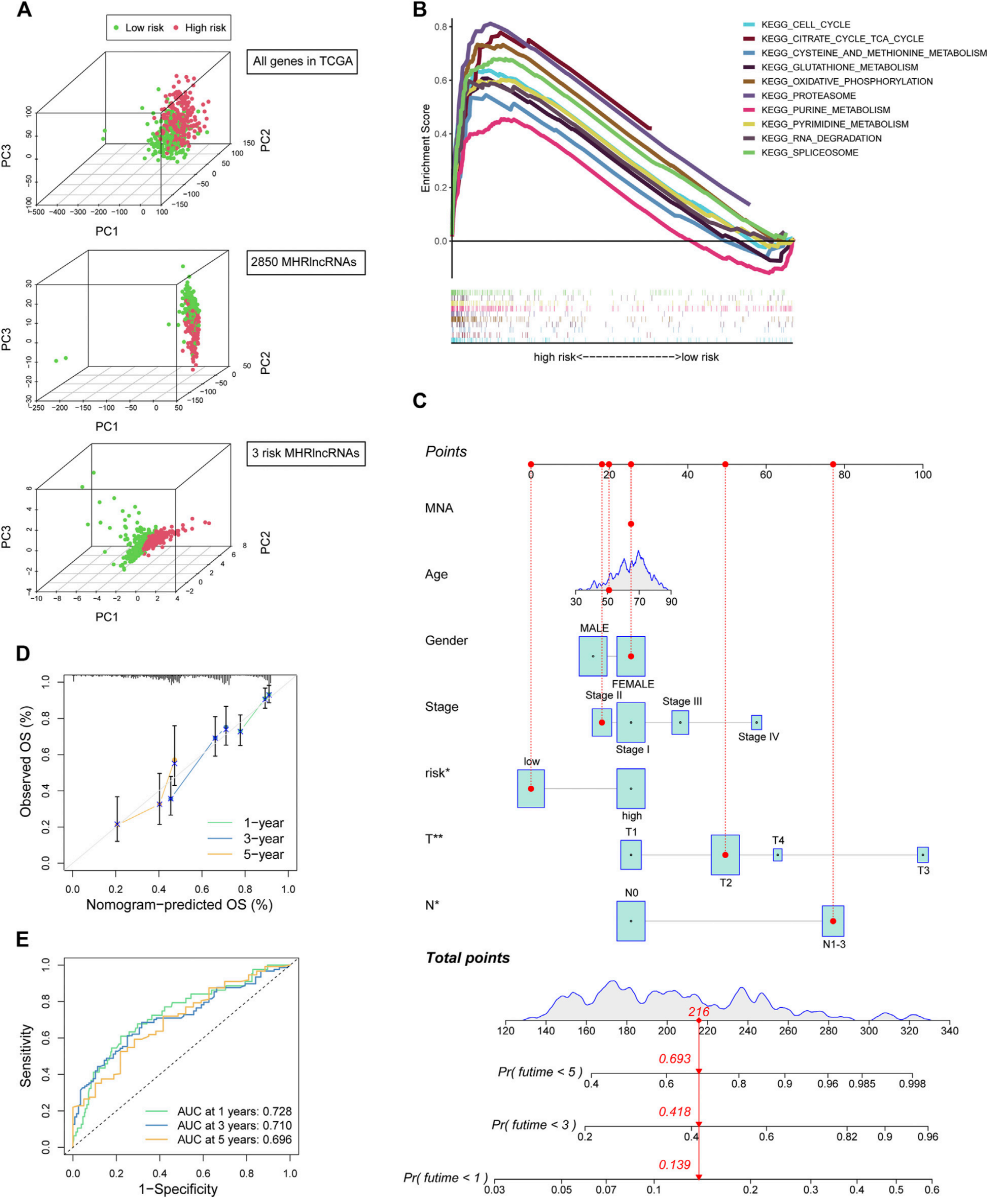
PCA and GSEA analyses of the MHLncSig and construction of a prognostic nomogram. **(A)** PCA based on the total gene expression profiles, 2,850 MHRlncRNAs, and the MHLncSig categorized by three risk MHRlncRNAs. **(B)** GSEA analysis of the high-risk group calculated by MHLncSig. **(C)** A nomogram predicting 1-, 2-, and 3-year OS of LUAD cancer. **(D)** The calibration plot of the nomogram. **(E)** ROC curves of the prognostic nomogram at 1, 3, and 5 years.

We conducted the PCA analysis to estimate the distributions of the two different risk groups based on the total gene expression profiles, 2,850 MHRlncRNAs, and the MHLncSig categorized by the expression profiles of the three risk lncRNAs (Figure 4A). Compared with the relatively scattered distributions of the two different risk groups based on the total gene expression profiles and 2,850 MHRlncRNAs, the results based on our prognostic signature indicated that the MHLncSig had excellent grouping ability to a certain extent. Furthermore, we performed GSEA enrichment analysis to reveal the underlying mechanisms and pathways behind the high-risk group characterized by dismal prognosis. A total of 37 pathways were significantly enriched in the high-risk group (FDR < 0.05, Supplementary Table S3). The pathways involving cell cycle, cysteine and methionine metabolism, glutathione metabolism, oxidative phosphorylation, proteasome, purine metabolism, and pyrimidine metabolism are visualized in Figure 4B.

### Establishment and Performance Evaluation of the Prognostic Nomogram

We constructed a prognostic nomogram comprising the risk signature and clinical traits to predict the survival rate of 1, 2, and 3 years (Figure 4C). A total score was assigned to each LUAD patient by combining six individual scores in the nomogram, where a lower total point was related to a better outcome. Furthermore, we utilized ROC curve and calibration plot analyses to evaluate the validity of the nomogram (Figures 4D,E). The prediction curve was very close to the actual survival curve in the calibration plot and the AUCs of 1-, 2-, and 3-year curve were 0.728, 0.71, and 0.696, respectively, suggesting that the nomogram predicted the prognosis for LUAD patients well.

### Evaluation of Tumor Microenvironment and TMB Using MHLncSig

**FIGURE 5:**
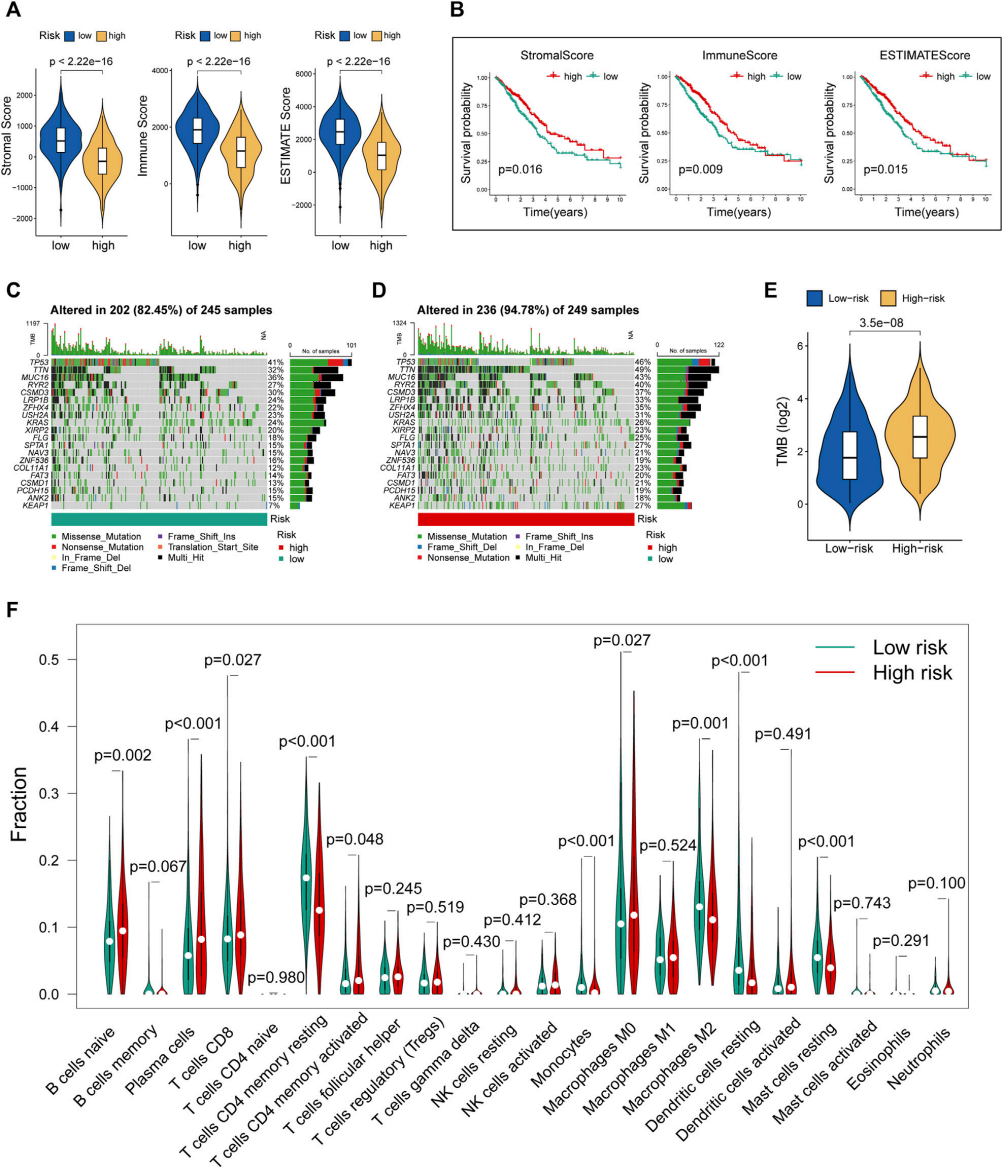
Evaluation of tumor microenvironment, TMB, and immune cell infiltration between different risk groups. **(A)** The differences of stromal score, immune score, and ESTIMATE score between the low- and high-risk groups. **(B)** The survival differences between the LUAD subgroups classified by stromal score, immune score, and ESTIMATE score. **(C)** The mutational landscape of the top 20 genes with the highest mutation frequency in the low-risk group. **(D)** The mutational landscape of the top 20 genes with the highest mutation frequency in the high-risk group. **(E)** The differences of TMB score between the low- and high-risk groups. **(F)** Estimation of immune cell infiltration in LUAD patients using the CIBERSORT algorithm.

Tumor microenvironment comprising stromal and immune components together with the secreted factors provide an immunosuppressive and protumoral environment for tumor development, which also correlates closely with immunotherapy positive response (Fridman et al., 2012; Gajewski et al., 2013). We made a comparison of the levels of stromal score, immune score, and ESTIMATE score between the two risk groups. The violin plots (Figure 5A) showed that all these three indicators were significantly decreased in the group with high risk. Further survival analysis suggested that lower levels of stromal score, immune score, and ESTIMATE score were significantly correlated with the worse prognosis in LUAD cohorts (Figure 5B). Moreover, we utilized R “maftools” package to analyze and visualize the mutational landscape of LUAD patients. The top 20 genes with the highest mutation frequency between the two risk subgroups are shown in Figures 5C,D. It is worth noting that the TMB score in the high-risk group exceeded that in the low-risk group, suggesting that MHLncSig had a high degree of TMB relevance (Figure 5E).

### Immune Landscape and Immunotherapeutic Response Based on the Prognostic Signature

**FIGURE 6:**
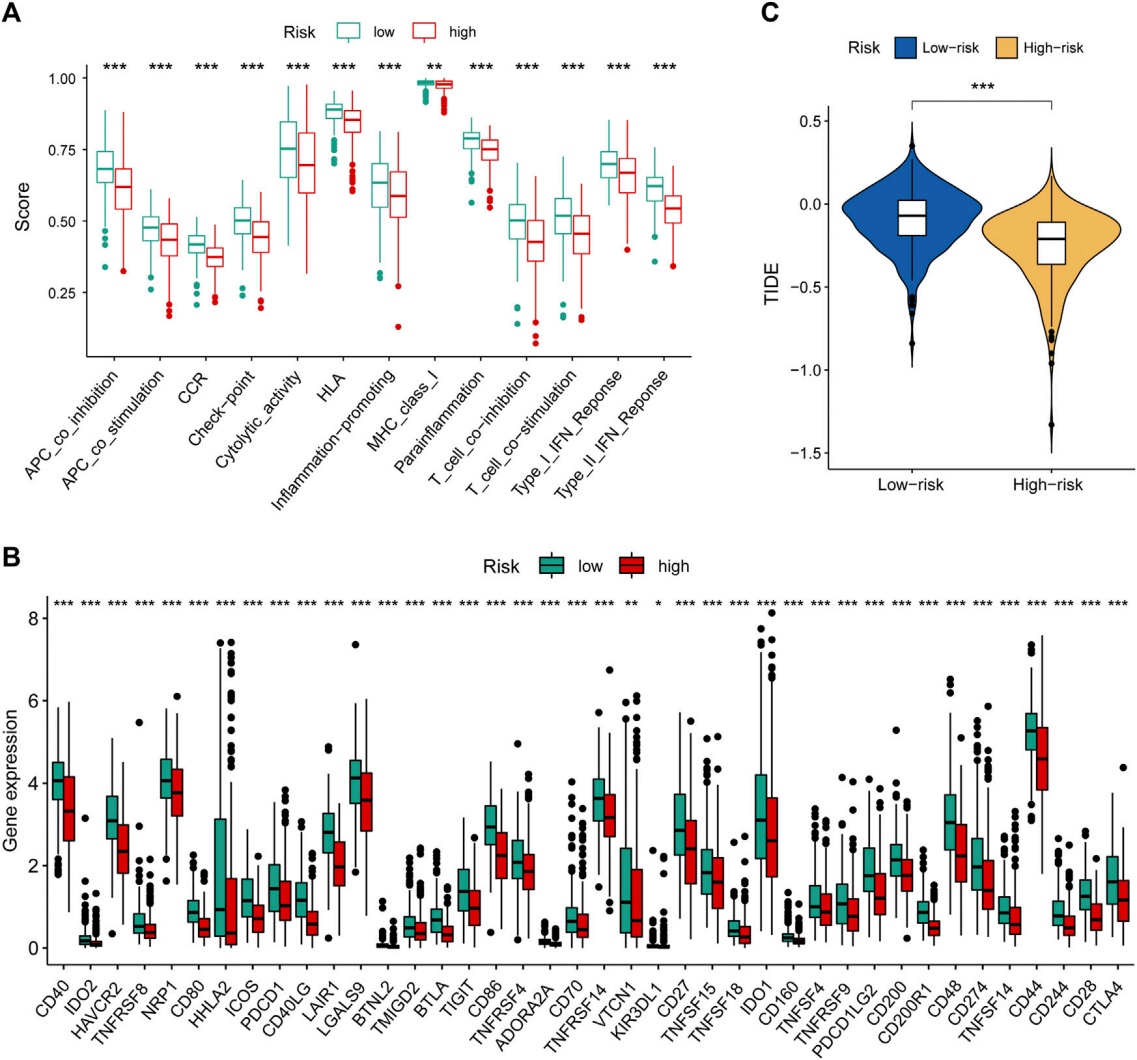
Immune landscape and immunotherapeutic response based on the MHLncSig. **(A)** The difference of immune function between the low- and high-risk groups. **(B)** Expression levels of immune checkpoint genes in the low- and high-risk patients. **(C)** TIDE difference between the low- and high-risk groups.

To further explore the relationship between our prognostic signature and immune infiltration, we used the CIBERSORT algorithm to analyze the relative abundance of 22 TICs in each LUAD patient. Figure 5F showed that the contents of nearly half of these TICs were significantly different between the two different risk groups. Of these, the plasma cells, T-cell CD8, B-cell naive, macrophages M0, and T-cell CD4 memory activated were dramatically increased in the high-risk group, while monocytes, macrophages M2, T-cell CD4 memory resting, mast cells resting, and dendritic cells resting were significantly decreased in the high-risk group. Moreover, we observed a comprehensive suppression of immune functions in the high-risk group (Figure 6A). To elucidate the relationship between the MHLncSig and immune checkpoint genes, the expression of immune checkpoint genes were compared between the low-risk (*n* = 252) and high-risk (*n* = 252) groups. The results indicated that 40 types of immune checkpoint genes were significantly upregulated in the low-risk group (Figure 6B). Previous literatures have reported that the TIDE algorithm is a powerful tool to assess the immunotherapeutic response (Jiang et al., 2018). We discovered that LUAD patients in the high-risk group are more likely to benefit from immunotherapy, indicating that the MHLncSig may serve as a potential indicator for predicting immunotherapeutic response (Figure 6C).

### Screening of Candidate Compounds Targeting MHLncSig and Model Validation in Human Surgical Resection Specimens

**FIGURE 7:**
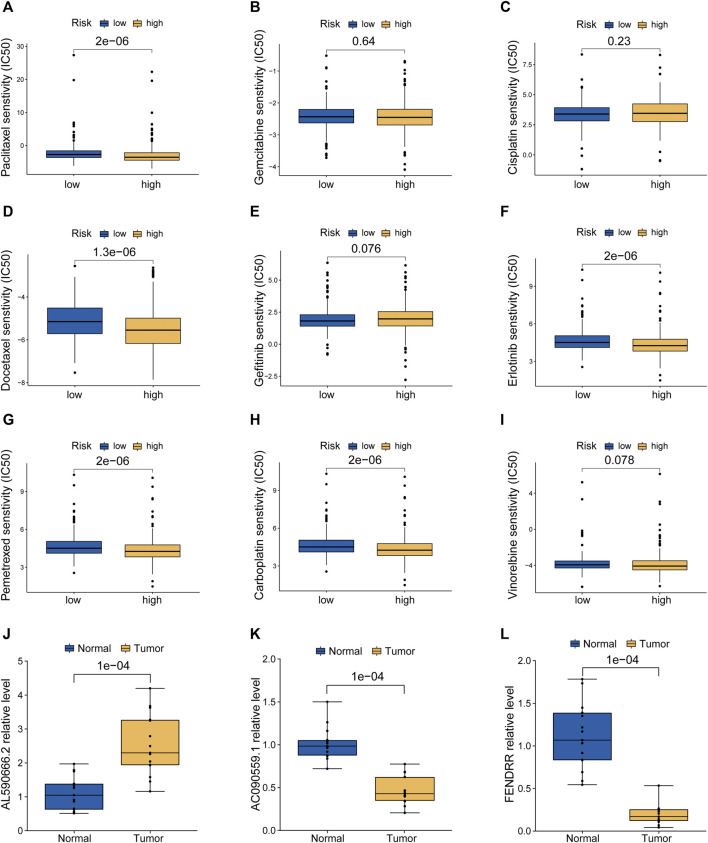
Screening of candidate compounds and model validation in human LUAD specimens. **(A–I)** Sensitivity analysis of nine chemotherapeutic and molecular-targeted agents in the low- and high-risk patients. **(J–L)** Validation of the expression differences of three risk MHRlncRNAs in human LUAD tissues and adjacent normal tissues.

According to the pRRophetic algorithm, we calculated the IC50 of nine chemotherapeutic and targeted agents in the low- and high-risk patients, which are recommended by the National Comprehensive Cancer Network (NCCN) guidelines for LUAD treatment. Wilcoxon test analysis indicated that five of these agents (paclitaxel, docetaxel, erlotinib, pemetrexed, and carboplatin) had lower IC50 in high-risk patients (*p* < 0.05, Figures 7A–I), which suggested that LUAD patients with high risk were more sensitive to these five agents. Finally, based on human paired LUAD tissues obtained by surgery, we validated the differential expression of three risk lncRNAs included in the MHLncSig by qRT-PCR assays (Figures 7J–L). The differential analysis revealed that AL590666.2 was significantly upregulated in LUAD tissues, while FENDRR and AC090559.1 were remarkably downregulated in LUAD tissues.

## Discussion

In actual clinical work, pathologic staging is still the mainstream procedure to predict long-term survival and guide treatment modality for LUAD patients. However, a population with the same pathologic staging would also present with distinct clinical outcomes, indicating that the traditional staging system could not accommodate the individualized prediction and treatment. More recently, next-generation sequencing brings a revolutionary change in predicting the prognosis of cancer (Nair et al., 2018). A growing number of prognostic signatures on the basis of encoding genes and noncoding RNAs were established to forecast the survival outcome of cancer patients (Peng et al., 2021; Zhou et al., 2021). To the best of our knowledge, we are the first to identify the prognostic MHLncSig and comprehensively analyze the tumor immunity of this risk model in LUAD, which provides valuable novel options for the guidance of clinical personalized treatment.

Due to their pivotal functions in the resistance of cancer cells to regulated cell death (RCD) induced by treatment, mitochondria have drawn considerable attention for their potential value in developing novel anticancer agents. Actually, mitochondria affect immunosurveillance through both the malignant cell-extrinsic and malignant cell-intrinsic mechanisms. From one side, the mitochondria of malignant cells release many danger signals when RCD occurs, and these signals are paramount for activating the dendritic cells to promote antitumor immune responses. From another aspect, mitochondrial metabolism involves a number of functions related to antitumor immunity, including the establishment of protective immunological memory, inflammasome activation, and the differentiation of macrophage subsets (Porporato et al., 2018). It is worth noting that dysregulation of mitochondrial metabolism is found to be associated with metastatic cascade and worse prognosis in the nine types of tumors (Gaude and Frezza, 2016). Moreover, the dysbiosis of mitochondrial homeostasis is related to the excessive generation of reactive oxygen species (ROS) and consequent metastatic cascade (Fu et al., 2017). Recently, accumulated evidence has suggested that lncRNAs play important roles in tumorigenesis and tumor development. The dysfunctional lncRNAs may be presumed as key factors in the active phases of tumors (Guan et al., 2019). Zheng et al. (2015) discovered novel mechanisms of lncRNA HOTAIR in maintaining mitochondrial function in the HeLa cells. In addition, nuclear-encoded lncRNA MALAT1 was reported to regulate the metabolic reprogramming through the mitophagy pathway in hepatocellular carcinoma cells (Zhao et al., 2021). Nonetheless, prognostic biomarkers of LUAD and studies on the protumor mechanisms concerning MHRlncRNAs are still lacking. Therefore, we attempted to establish an independent signature for predicting the prognosis of LUAD based on MHRlncRNAs.

In our study, we identified 2,850 MHRlncRNAs based on the TCGA data. Subsequent differential analysis and univariate Cox analysis confirmed nine MHRlncRNAs with prognostic value, and three of these were utilized to construct a MHLncSig to predict the OS of LUAD patients.

Among them, FENDRR is commonly considered to be a tumor suppressor, which has been extensively studied in multiple cancer types, including cholangiocarcinoma, malignant melanoma, non–small cell lung cancer, hepatocellular carcinoma, and breast cancer (Li et al., 2018b; Zhang et al., 2018; Wang et al., 2019b; Qin et al., 2019; Chen et al., 2020). Nevertheless, specific mechanisms of two other lncRNAs (AL590666.2 and AC090559.1) during tumorigenesis and progression have never been previously reported and our research unmasked their underlying roles in LUAD.

TMB is defined typically as the total number of somatic coding mutations, which is associated with the production of neoantigens that induce antitumor immunity (Allgäuer et al., 2018). Recent studies have indicated that the higher TMB score was related to durable clinical benefit and improved objective response in tumor immunotherapy (Rizvi et al., 2015). Notably, the LUAD patients with high risk tend to possess higher levels of TMB, indicating that they are more likely to benefit from immunotherapy. This finding was subsequently confirmed by the TIDE algorithm. Moreover, another significant finding of our study suggested that the MHLncSig was significantly associated with the immune infiltration in LUAD, which further supported the fact that mitochondrial homeostasis plays a critical role in the tumor immune microenvironment.

There are still several limitations of the present study that need to be considered. First, it would be better to validate our prognostic model with several external datasets. In fact, we were unable to find an ideal Gene Expression Omnibus (GEO) database owing to the lack of complete expression profiles of the three risk MHRlncRNAs and detailed clinical information. Second, although we have initially screened a number of candidate compounds targeting MHLncSig in our study, the sensitivity of LUAD patients to specific immunotherapy agents requires further investigation due to a lack of immunotherapy drugs for LUAD in the R “pRRophetic” package.

Finally, functional experiments should be performed *in vivo* and *in vitro* to further corroborate our findings.

In summary, using multiple bioinformatics approaches and qRT-PCR experiments, we established and validated a 3-MHRlncRNA signature to independently predict the OS of LUAD patients. In addition, the MHLncSig shows advantages in terms of guiding individualized treatment of LUAD.

## Conclusion

In conclusion, we successfully developed an accurate prognostic model of mitochondrial homeostasis, which was closely associated with tumor microenvironment, TMB, immune infiltration, and the response to immunotherapy in LUAD. Meanwhile, the findings in our study may provide clues for further elucidating the molecular mechanism of MHRlncRNAs in the tumorigenesis and LUAD progression.

## Data Availability

The datasets presented in this study can be found in online repositories. The names of the repository/repositories and accession number(s) can be found in the article/Supplementary Material.
