# Supplementary material for: Mitochondrial Homeostasis–Related lncRNAs are Potential Biomarkers for Predicting Prognosis and Immune Response in Lung Adenocarcinoma
Source: Front Genet. 2022 Jun 13;13:870302. doi: 10.3389/fgene.2022.870302 (PMC9234294; doi:10.3389/fgene.2022.870302)
Supplement: Supplementary file 1 [file DataSheet1.docx]

**Supplementary Materials**

**
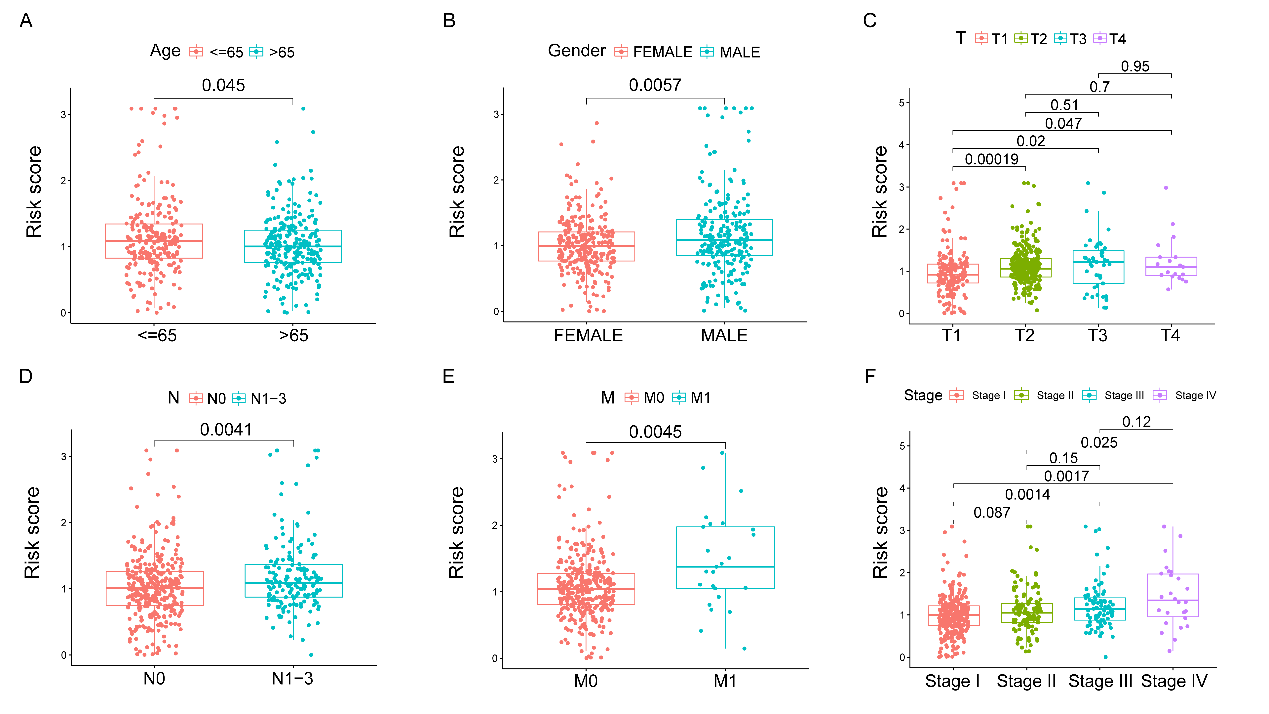
**

**Supplementary Figure S1.** Correlation analysis between the MHLncSig and clinical traits in LUAD.

**
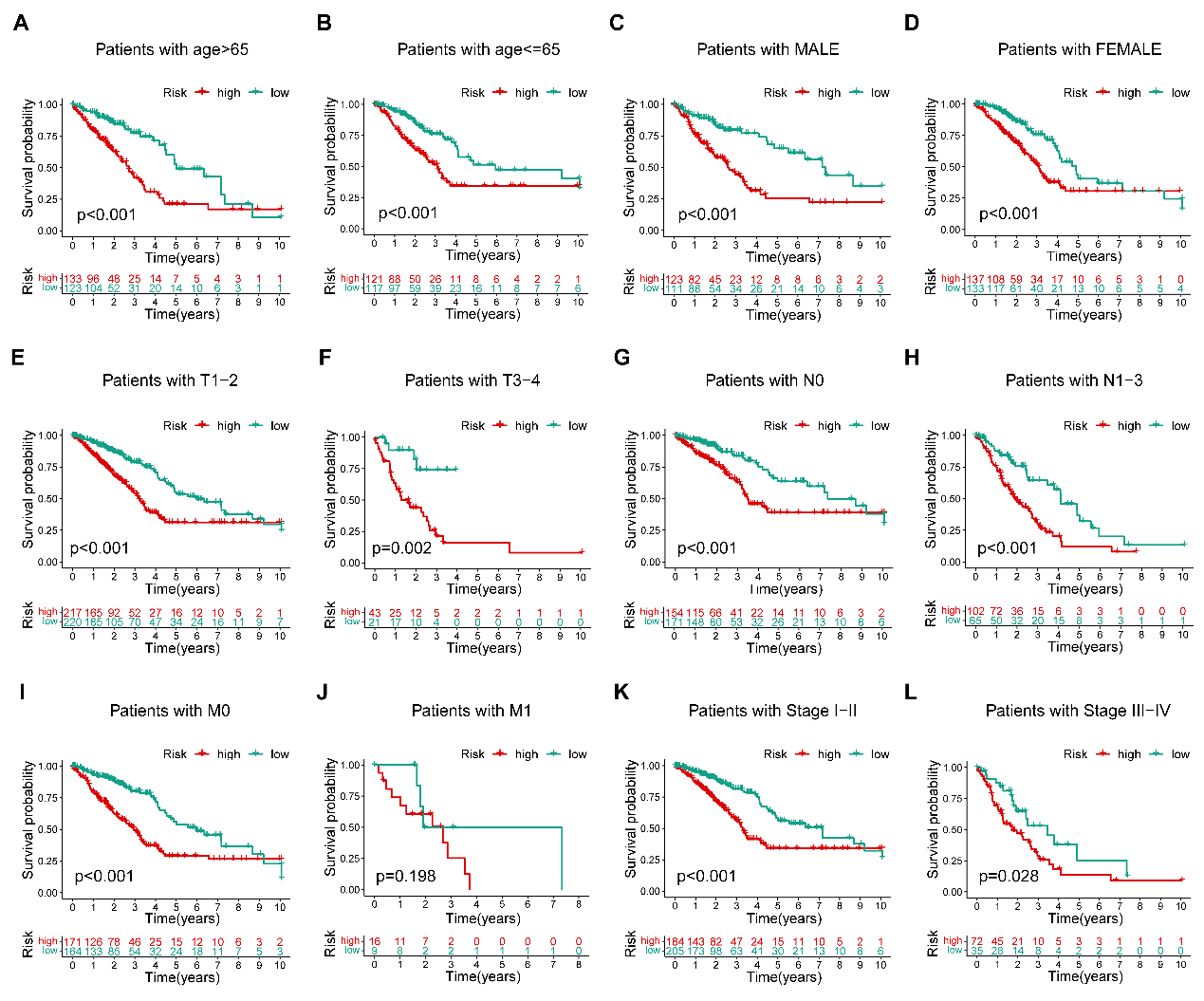
**

**Supplementary Figure S2.** Performance evaluation of our MHLncSig by Stratification analyses in different subgroups stratified by age **(A, B)**, gender **(C, D)**, T stage **(E, F)**, N stage **(G, H)**, M stage **(I, J)**, and pathologic stage **(K, L)**.

**Supplementary Table S1.** 294 differentially expressed genes related to mitochondrial homeostasis between tumor and normal samples.

| gene | conMean | treatMean | logFC | pValue | FDR |
| --- | --- | --- | --- | --- | --- |
| TWNK | 1.713036 | 4.448082 | 1.376628 | 6.82E-28 | 8.79E-27 |
| POLG2 | 1.463996 | 3.803633 | 1.377466 | 4.68E-27 | 4.90E-26 |
| TRMU | 1.45254 | 3.031517 | 1.061462 | 2.27E-22 | 1.31E-21 |
| MT-TE | 0.755247 | 0.287775 | -1.39201 | 1.06E-10 | 2.31E-10 |
| NDUFS6 | 21.28542 | 44.82857 | 1.074553 | 1.05E-22 | 6.21E-22 |
| MGME1 | 4.694798 | 9.649817 | 1.039439 | 2.53E-28 | 3.61E-27 |
| NDUFAF6 | 1.510571 | 3.18281 | 1.075207 | 9.40E-21 | 4.49E-20 |
| MSTO1 | 1.305528 | 4.021148 | 1.622974 | 2.03E-34 | 2.38E-32 |
| MT-TV | 0.343102 | 1.71539 | 2.321829 | 0.032893 | 0.037617 |
| BOLA3 | 2.287305 | 4.64278 | 1.02134 | 5.46E-22 | 2.97E-21 |
| UQCC2 | 3.220863 | 9.735275 | 1.595774 | 4.57E-31 | 1.18E-29 |
| MRPL12 | 9.588557 | 24.21286 | 1.336388 | 2.42E-23 | 1.55E-22 |
| TSFM | 5.103365 | 10.36952 | 1.022829 | 4.14E-27 | 4.38E-26 |
| CYC1 | 28.75142 | 60.42947 | 1.071619 | 3.21E-20 | 1.44E-19 |
| SLC25A10 | 1.46715 | 7.255367 | 2.306032 | 1.61E-31 | 4.84E-30 |
| DNA2 | 0.389165 | 2.046537 | 2.394733 | 7.67E-32 | 2.54E-30 |
| UQCC3 | 4.250509 | 9.65516 | 1.183664 | 3.82E-23 | 2.42E-22 |
| PUS1 | 1.758524 | 4.37879 | 1.316167 | 4.14E-27 | 4.38E-26 |
| TIMM8A | 1.244792 | 3.16376 | 1.345736 | 3.70E-32 | 1.36E-30 |
| DARS2 | 4.148473 | 10.67479 | 1.363555 | 3.38E-30 | 6.81E-29 |
| GAREM2 | 0.381506 | 1.636993 | 2.101271 | 2.28E-21 | 1.16E-20 |
| FANCI | 0.978002 | 4.305453 | 2.138255 | 2.86E-33 | 2.05E-31 |
| MT-TS2 | 0.262889 | 0.635437 | 1.273293 | 0.001699 | 0.002164 |
| SLC25A21 | 0.098201 | 0.500568 | 2.349756 | 7.41E-11 | 1.64E-10 |
| COA6 | 8.563693 | 20.88509 | 1.286169 | 5.61E-25 | 4.36E-24 |
| MRPL3 | 15.91515 | 35.90047 | 1.173602 | 3.33E-32 | 1.34E-30 |
| TARS2 | 4.750961 | 10.74206 | 1.176979 | 5.51E-33 | 3.01E-31 |
| MARS2 | 1.734797 | 3.553776 | 1.034586 | 4.79E-25 | 3.75E-24 |
| MRPL36 | 6.665575 | 15.15531 | 1.185022 | 1.16E-24 | 8.88E-24 |
| HSPD1 | 31.14984 | 83.49159 | 1.422406 | 7.94E-33 | 3.94E-31 |
| CKMT1B | 0.124636 | 0.970507 | 2.961022 | 1.63E-12 | 4.08E-12 |
| SARS2 | 0.975459 | 2.015608 | 1.047062 | 9.93E-19 | 4.04E-18 |
| SLC25A13 | 4.651836 | 10.82448 | 1.218426 | 3.39E-29 | 5.54E-28 |
| CKMT1A | 0.115329 | 0.862296 | 2.902424 | 4.37E-13 | 1.15E-12 |
| MRPL13 | 4.633364 | 9.353337 | 1.013421 | 3.10E-21 | 1.56E-20 |
| ATAD3A | 3.682977 | 9.278139 | 1.332963 | 1.13E-27 | 1.37E-26 |
| PPARG | 12.49131 | 4.583032 | -1.44655 | 1.03E-25 | 8.58E-25 |
| ALDH2 | 51.56547 | 24.43306 | -1.07757 | 6.98E-26 | 6.05E-25 |
| PARS2 | 1.487179 | 3.132788 | 1.074869 | 3.42E-27 | 3.71E-26 |
| IL6 | 26.9944 | 4.258953 | -2.66409 | 2.68E-14 | 7.68E-14 |
| MRPL15 | 19.26583 | 45.9895 | 1.25526 | 7.22E-26 | 6.21E-25 |
| SNCA | 1.880944 | 0.741169 | -1.34358 | 3.54E-26 | 3.23E-25 |
| MRPL24 | 17.04964 | 39.0902 | 1.197066 | 1.67E-27 | 1.92E-26 |
| SLC39A8 | 99.29098 | 19.51687 | -2.34694 | 2.41E-35 | 4.44E-33 |
| MRPL9 | 14.27806 | 30.06216 | 1.074149 | 4.02E-30 | 7.87E-29 |
| CYP27A1 | 69.03513 | 23.32754 | -1.5653 | 3.54E-28 | 4.81E-27 |
| CYP27B1 | 0.431851 | 2.429642 | 2.492137 | 5.47E-29 | 8.61E-28 |
| MTG1 | 0.837294 | 1.915773 | 1.19412 | 3.05E-20 | 1.38E-19 |
| SFXN4 | 5.1723 | 14.66166 | 1.503171 | 4.29E-33 | 2.56E-31 |
| TOP1MT | 2.609473 | 5.363497 | 1.039416 | 6.84E-21 | 3.33E-20 |
| PGAM5 | 6.624605 | 14.46109 | 1.12627 | 3.27E-26 | 3.04E-25 |
| ALAS2 | 1.361211 | 0.094866 | -3.84285 | 7.46E-26 | 6.37E-25 |
| HJV | 0.012084 | 0.043447 | 1.846132 | 0.004457 | 0.005465 |
| ABCB6 | 0.458242 | 1.999519 | 2.12547 | 5.65E-30 | 1.08E-28 |
| ACADL | 6.679917 | 0.841702 | -2.98845 | 1.16E-34 | 1.49E-32 |
| DAP3 | 13.30665 | 27.13381 | 1.027945 | 1.44E-32 | 6.87E-31 |
| MTARC2 | 17.07112 | 6.791563 | -1.32974 | 9.44E-31 | 2.30E-29 |
| SLC2A1 | 3.128873 | 38.73479 | 3.629915 | 3.56E-32 | 1.36E-30 |
| COX4I2 | 25.85822 | 5.878556 | -2.13709 | 7.01E-31 | 1.74E-29 |
| PPIF | 10.53395 | 24.68052 | 1.228327 | 8.72E-28 | 1.09E-26 |
| SHMT2 | 7.959626 | 24.22738 | 1.605865 | 7.31E-32 | 2.48E-30 |
| SLC25A15 | 1.775496 | 3.767447 | 1.085365 | 4.77E-19 | 1.99E-18 |
| SLC25A25 | 14.26874 | 5.077625 | -1.49063 | 4.80E-20 | 2.11E-19 |
| LRRK2 | 30.71856 | 8.595666 | -1.83743 | 1.99E-26 | 1.88E-25 |
| CAT | 100.3874 | 29.61703 | -1.76108 | 1.95E-35 | 4.44E-33 |
| HAMP | 0.244425 | 0.640289 | 1.389332 | 6.97E-10 | 1.40E-09 |
| PDF | 0.679124 | 1.591907 | 1.22901 | 3.95E-23 | 2.48E-22 |
| OTC | 0.623361 | 0.054325 | -3.52038 | 4.27E-33 | 2.56E-31 |
| SLC39A14 | 4.323341 | 8.734559 | 1.014588 | 1.97E-11 | 4.53E-11 |
| GSR | 19.50857 | 42.36083 | 1.118622 | 5.35E-11 | 1.20E-10 |
| PARP1 | 13.51054 | 28.90555 | 1.097261 | 5.56E-28 | 7.25E-27 |
| CYP24A1 | 0.238845 | 25.60108 | 6.743988 | 1.45E-27 | 1.71E-26 |
| TRAP1 | 5.074658 | 12.33505 | 1.281381 | 3.99E-32 | 1.43E-30 |
| GAMT | 3.25835 | 7.767479 | 1.253305 | 9.42E-12 | 2.23E-11 |
| WFS1 | 41.98495 | 13.83234 | -1.60183 | 4.69E-32 | 1.63E-30 |
| GARS1 | 15.03615 | 34.33498 | 1.191244 | 2.37E-31 | 6.66E-30 |
| CP | 4.752815 | 57.22063 | 3.589681 | 4.97E-23 | 3.08E-22 |
| BNIP3 | 7.395377 | 17.84547 | 1.270862 | 1.19E-20 | 5.63E-20 |
| PC | 1.959748 | 7.027951 | 1.842436 | 1.40E-27 | 1.67E-26 |
| MTHFD2 | 5.908016 | 18.69243 | 1.661708 | 3.75E-24 | 2.67E-23 |
| ALB | 0.058103 | 29.99037 | 9.011677 | 0.013388 | 0.015888 |
| OAT | 20.26939 | 43.69947 | 1.108313 | 7.44E-15 | 2.22E-14 |
| IDH2 | 23.17755 | 57.79848 | 1.318304 | 1.81E-26 | 1.72E-25 |
| XDH | 0.236333 | 4.310917 | 4.189103 | 3.48E-28 | 4.77E-27 |
| SLC8A3 | 0.168623 | 0.06635 | -1.34563 | 6.10E-21 | 2.99E-20 |
| PRKAA2 | 0.638836 | 1.655381 | 1.373646 | 1.90E-07 | 3.22E-07 |
| PDK4 | 53.4055 | 11.53916 | -2.21045 | 1.54E-27 | 1.81E-26 |
| EPRS1 | 16.66953 | 34.52246 | 1.050322 | 8.58E-29 | 1.32E-27 |
| CD36 | 19.92681 | 2.925336 | -2.76804 | 5.72E-33 | 3.01E-31 |
| VARS1 | 9.290166 | 23.0307 | 1.309782 | 2.24E-29 | 3.90E-28 |
| MYH7 | 0.004643 | 0.036529 | 2.975991 | 2.49E-08 | 4.48E-08 |
| GHR | 1.403242 | 0.393606 | -1.83394 | 6.25E-30 | 1.14E-28 |
| CPOX | 3.632833 | 7.519834 | 1.049606 | 2.13E-28 | 3.13E-27 |
| PDK1 | 0.666273 | 2.481857 | 1.897234 | 1.33E-30 | 3.12E-29 |
| HNF1A | 0.034059 | 0.51051 | 3.905821 | 2.15E-05 | 3.13E-05 |
| CFTR | 5.337598 | 2.554318 | -1.06325 | 1.05E-16 | 3.66E-16 |
| SLC6A3 | 0.134945 | 2.324019 | 4.106184 | 0.001686 | 0.002151 |
| HMOX1 | 77.18324 | 27.76545 | -1.475 | 4.41E-07 | 7.34E-07 |
| SLC19A3 | 5.175614 | 0.759843 | -2.76796 | 2.53E-32 | 1.11E-30 |
| GPT | 0.297428 | 1.295157 | 2.122513 | 1.88E-11 | 4.32E-11 |
| BOLA1 | 5.561358 | 11.74331 | 1.078329 | 3.05E-20 | 1.38E-19 |
| GDF15 | 15.30221 | 46.21328 | 1.594568 | 5.75E-06 | 8.69E-06 |
| CA5A | 0.008306 | 0.038347 | 2.206951 | 1.22E-10 | 2.63E-10 |
| LEPR | 6.491099 | 1.984133 | -1.70995 | 2.88E-29 | 4.89E-28 |
| NME4 | 9.012169 | 25.8016 | 1.517514 | 8.95E-27 | 8.88E-26 |
| GJB2 | 0.644261 | 16.33446 | 4.66413 | 4.57E-31 | 1.18E-29 |
| MTX1 | 3.391586 | 6.834189 | 1.01081 | 2.31E-28 | 3.35E-27 |
| AK4 | 0.595692 | 3.911366 | 2.715035 | 7.20E-19 | 2.98E-18 |
| SLC12A3 | 0.043674 | 0.131953 | 1.595171 | 0.001178 | 0.00152 |
| CDKN2A | 0.498778 | 6.306165 | 3.660292 | 1.45E-14 | 4.25E-14 |
| TFR2 | 0.085558 | 0.792223 | 3.210934 | 4.37E-33 | 2.56E-31 |
| CAV3 | 0.386395 | 0.053907 | -2.84154 | 1.29E-30 | 3.07E-29 |
| PIF1 | 0.172689 | 0.980296 | 2.505039 | 6.98E-26 | 6.05E-25 |
| ACE | 14.86398 | 4.925422 | -1.5935 | 5.67E-29 | 8.82E-28 |
| COA4 | 11.16001 | 22.59625 | 1.017745 | 5.66E-27 | 5.70E-26 |
| ATAD3B | 1.296969 | 3.954544 | 1.608368 | 1.90E-23 | 1.24E-22 |
| HSPE1 | 17.54837 | 46.41856 | 1.403364 | 6.08E-30 | 1.12E-28 |
| ABCG1 | 16.80067 | 7.13291 | -1.23596 | 1.64E-26 | 1.56E-25 |
| RETN | 28.16356 | 4.006318 | -2.81348 | 6.02E-30 | 1.12E-28 |
| FLAD1 | 4.969548 | 14.33484 | 1.528339 | 1.06E-33 | 8.06E-32 |
| CTLA4 | 0.97538 | 2.222658 | 1.188249 | 2.14E-09 | 4.15E-09 |
| AQP4 | 106.6203 | 15.23792 | -2.80674 | 9.04E-35 | 1.30E-32 |
| TNFSF11 | 0.056618 | 1.020431 | 4.171761 | 1.24E-24 | 9.46E-24 |
| DMGDH | 0.183622 | 0.693566 | 1.917297 | 0.000121 | 0.000168 |
| FOXP3 | 0.604574 | 2.389851 | 1.98293 | 1.55E-23 | 1.03E-22 |
| CAV1 | 344.7297 | 34.83055 | -3.30704 | 1.09E-35 | 4.44E-33 |
| PCSK9 | 7.800324 | 3.012647 | -1.3725 | 1.38E-22 | 8.05E-22 |
| LDLR | 52.97931 | 13.71855 | -1.9493 | 1.15E-28 | 1.75E-27 |
| CNR1 | 2.43322 | 0.965062 | -1.33417 | 9.45E-19 | 3.86E-18 |
| HSPA5 | 126.0601 | 255.2033 | 1.017536 | 5.09E-29 | 8.11E-28 |
| SAMMSON | 0.004536 | 0.039292 | 3.114802 | 6.32E-08 | 1.11E-07 |
| HBB | 616.2297 | 36.63233 | -4.07228 | 9.35E-32 | 3.02E-30 |
| SLC34A1 | 0.01135 | 0.030305 | 1.416802 | 4.50E-11 | 1.01E-10 |
| DHTKD1 | 4.466687 | 10.07277 | 1.173183 | 2.23E-27 | 2.52E-26 |
| SLC13A5 | 0.015926 | 0.184528 | 3.534406 | 4.55E-05 | 6.53E-05 |
| TERT | 0.004868 | 0.250591 | 5.685889 | 4.03E-31 | 1.08E-29 |
| SLC25A39 | 21.08791 | 53.31503 | 1.338126 | 2.94E-30 | 6.13E-29 |
| PTRH2 | 2.439905 | 5.364446 | 1.136604 | 2.59E-30 | 5.47E-29 |
| NAXE | 29.47558 | 67.52569 | 1.195916 | 4.56E-24 | 3.23E-23 |
| AGT | 1.646394 | 15.75875 | 3.258771 | 5.98E-08 | 1.05E-07 |
| SPATA18 | 7.066711 | 2.87762 | -1.29616 | 5.63E-13 | 1.45E-12 |
| MMACHC | 0.939576 | 2.108532 | 1.166157 | 2.36E-30 | 5.25E-29 |
| FLVCR1 | 1.588374 | 4.166082 | 1.391141 | 3.92E-25 | 3.09E-24 |
| TH | 0.018362 | 0.097376 | 2.406828 | 0.024549 | 0.028402 |
| SFTPC | 7909.18 | 334.8571 | -4.56191 | 5.63E-36 | 3.63E-33 |
| PPARGC1B | 1.129739 | 0.475243 | -1.24925 | 1.29E-19 | 5.53E-19 |
| IFNG | 0.329487 | 0.869432 | 1.399853 | 0.009089 | 0.010917 |
| PDX1 | 0.001 | 0.594199 | 9.214989 | 7.12E-13 | 1.83E-12 |
| LPL | 51.71286 | 13.20529 | -1.96941 | 3.13E-28 | 4.34E-27 |
| ALDH1L2 | 0.719084 | 1.784435 | 1.311235 | 5.69E-07 | 9.40E-07 |
| IL2RA | 1.207109 | 3.618514 | 1.583842 | 2.09E-14 | 6.05E-14 |
| ABCA4 | 0.207503 | 2.972594 | 3.840516 | 4.63E-09 | 8.73E-09 |
| BIRC5 | 0.806704 | 11.4556 | 3.82787 | 2.17E-32 | 9.99E-31 |
| ACAD8 | 3.208693 | 8.737918 | 1.445304 | 2.21E-20 | 1.02E-19 |
| RPE65 | 0.00858 | 0.074699 | 3.122014 | 0.014192 | 0.016781 |
| HMGA1 | 22.9883 | 122.422 | 2.412891 | 2.54E-30 | 5.46E-29 |
| DNM1 | 0.668171 | 1.626114 | 1.283138 | 3.59E-11 | 8.14E-11 |
| NOS1 | 0.896052 | 0.254531 | -1.81574 | 5.51E-27 | 5.64E-26 |
| SCN4A | 0.030154 | 0.332248 | 3.461832 | 4.84E-08 | 8.57E-08 |
| EGF | 0.341358 | 1.796843 | 2.396107 | 1.41E-13 | 3.87E-13 |
| KL | 3.741415 | 0.827148 | -2.17737 | 4.92E-31 | 1.25E-29 |
| GAPDH | 216.9479 | 770.9202 | 1.829233 | 3.29E-30 | 6.73E-29 |
| SLC4A1 | 0.189787 | 0.020536 | -3.20816 | 8.01E-23 | 4.80E-22 |
| MMP1 | 1.59358 | 64.37618 | 5.336184 | 3.79E-25 | 3.00E-24 |
| NEXN | 7.549117 | 2.906434 | -1.37706 | 1.51E-24 | 1.13E-23 |
| SFXN1 | 2.175719 | 6.794359 | 1.642845 | 2.14E-35 | 4.44E-33 |
| MB | 0.496206 | 4.88369 | 3.298961 | 3.40E-21 | 1.70E-20 |
| KCNJ5 | 4.419824 | 1.423365 | -1.63468 | 4.41E-26 | 3.98E-25 |
| PMAIP1 | 2.999817 | 10.39457 | 1.792884 | 2.62E-14 | 7.51E-14 |
| NEB | 0.061601 | 0.280646 | 2.187736 | 1.70E-06 | 2.67E-06 |
| PMM2 | 1.221376 | 3.000484 | 1.296688 | 1.53E-31 | 4.69E-30 |
| GLDC | 0.180111 | 1.448384 | 3.007482 | 3.07E-09 | 5.88E-09 |
| H2AC7 | 0.124034 | 3.41957 | 4.785003 | 1.21E-13 | 3.33E-13 |
| TF | 0.212984 | 5.758721 | 4.756934 | 0.002603 | 0.003279 |
| CPT1B | 0.182946 | 0.755402 | 2.045825 | 4.16E-18 | 1.63E-17 |
| TMPRSS6 | 0.660475 | 3.501037 | 2.406207 | 0.013126 | 0.015606 |
| MAOB | 18.54889 | 7.109124 | -1.38359 | 8.93E-26 | 7.58E-25 |
| BMP2 | 22.65256 | 7.36702 | -1.62052 | 1.39E-23 | 9.32E-23 |
| MYH6 | 0.000813 | 0.020221 | 4.636256 | 3.24E-13 | 8.66E-13 |
| CXCL12 | 14.45391 | 6.667421 | -1.11626 | 1.11E-20 | 5.28E-20 |
| PKM | 78.9756 | 161.822 | 1.034929 | 9.55E-26 | 8.00E-25 |
| ALDH1B1 | 6.133906 | 14.22436 | 1.213486 | 1.15E-21 | 6.03E-21 |
| TNNI3 | 0.238688 | 1.750981 | 2.874965 | 0.004633 | 0.005676 |
| BOLA2B | 0.18935 | 0.423278 | 1.160549 | 2.42E-13 | 6.52E-13 |
| CACNA1S | 0.220366 | 0.024544 | -3.16648 | 1.07E-29 | 1.92E-28 |
| AGRP | 4.688615 | 0.67612 | -2.79381 | 2.49E-30 | 5.45E-29 |
| SCN5A | 0.025061 | 0.101104 | 2.012305 | 0.022248 | 0.02581 |
| ARG2 | 1.229949 | 3.050276 | 1.310341 | 1.51E-09 | 2.95E-09 |
| C11orf65 | 0.131765 | 0.281669 | 1.096037 | 9.94E-07 | 1.59E-06 |
| PGR | 0.586146 | 0.202774 | -1.53139 | 3.29E-25 | 2.64E-24 |
| GCLC | 2.810025 | 14.63844 | 2.381107 | 1.30E-12 | 3.31E-12 |
| ATP2A1 | 0.099443 | 0.365266 | 1.877011 | 5.82E-18 | 2.25E-17 |
| APLN | 12.10702 | 4.227025 | -1.51813 | 2.76E-15 | 8.49E-15 |
| BDNF | 1.412052 | 0.308034 | -2.19663 | 4.34E-28 | 5.72E-27 |
| CEP55 | 0.722848 | 7.684068 | 3.410107 | 5.84E-33 | 3.01E-31 |
| MC4R | 0.009064 | 0.076983 | 3.08638 | 6.63E-11 | 1.48E-10 |
| GCSH | 1.005736 | 2.230363 | 1.149027 | 2.18E-25 | 1.76E-24 |
| TTPA | 0.477535 | 0.186519 | -1.35628 | 1.21E-21 | 6.30E-21 |
| CDKN2B | 14.98922 | 6.770364 | -1.14662 | 4.77E-20 | 2.10E-19 |
| TYMS | 2.515166 | 10.35988 | 2.042282 | 3.10E-29 | 5.19E-28 |
| SCN1A | 1.215383 | 0.375359 | -1.69507 | 1.07E-23 | 7.22E-23 |
| CDK1 | 1.559918 | 9.505669 | 2.607318 | 3.27E-29 | 5.41E-28 |
| ACACB | 2.371783 | 1.053739 | -1.17045 | 2.05E-21 | 1.05E-20 |
| CYP2D6 | 0.244419 | 1.054763 | 2.109494 | 6.52E-11 | 1.45E-10 |
| KCNJ10 | 0.072913 | 0.359241 | 2.300699 | 3.93E-14 | 1.10E-13 |
| CYP2E1 | 0.092942 | 0.276996 | 1.575458 | 0.000652 | 0.000859 |
| BRCA1 | 0.689916 | 2.127065 | 1.624371 | 9.06E-20 | 3.90E-19 |
| IGFBP3 | 16.59999 | 81.76155 | 2.30024 | 4.54E-21 | 2.25E-20 |
| F2 | 0.004498 | 0.803637 | 7.481125 | 3.09E-09 | 5.92E-09 |
| CYP19A1 | 0.033011 | 0.096082 | 1.541319 | 6.80E-08 | 1.19E-07 |
| CPT1C | 0.415153 | 0.856715 | 1.045173 | 0.013274 | 0.015767 |
| KIF5A | 0.046902 | 0.338081 | 2.849649 | 6.48E-15 | 1.94E-14 |
| PYCR1 | 3.049044 | 43.9607 | 3.849785 | 2.07E-35 | 4.44E-33 |
| FGFR4 | 16.10981 | 3.229638 | -2.3185 | 1.38E-30 | 3.18E-29 |
| EPO | 0.021398 | 0.056921 | 1.411467 | 0.006263 | 0.007629 |
| GIMAP5 | 1.295716 | 0.486417 | -1.41348 | 4.89E-23 | 3.05E-22 |
| TBRG4 | 5.070502 | 11.76652 | 1.214488 | 1.91E-30 | 4.32E-29 |
| AVPR2 | 1.234203 | 0.358547 | -1.78335 | 1.39E-27 | 1.67E-26 |
| ATP6V0A4 | 0.524305 | 2.328738 | 2.15107 | 0.035812 | 0.04081 |
| TLR4 | 10.64883 | 4.105359 | -1.37511 | 2.10E-26 | 1.96E-25 |
| CYP7A1 | 0.041565 | 0.013993 | -1.57066 | 2.19E-19 | 9.28E-19 |
| CETP | 1.874524 | 0.86511 | -1.11557 | 6.88E-17 | 2.44E-16 |
| GCAT | 3.1456 | 7.433757 | 1.240756 | 3.00E-16 | 1.01E-15 |
| CHEK2 | 1.240853 | 3.807431 | 1.617485 | 7.44E-28 | 9.51E-27 |
| EDN1 | 47.06019 | 13.16776 | -1.8375 | 2.09E-22 | 1.21E-21 |
| POU6F2 | 0.036647 | 0.63639 | 4.118149 | 3.35E-05 | 4.86E-05 |
| TPD52 | 6.81715 | 15.86206 | 1.21834 | 6.09E-22 | 3.29E-21 |
| C1orf112 | 0.468137 | 1.748614 | 1.901208 | 2.86E-34 | 3.08E-32 |
| BCL2A1 | 20.93467 | 9.994504 | -1.06669 | 5.47E-16 | 1.81E-15 |
| AK1 | 15.21574 | 7.210308 | -1.07743 | 3.03E-24 | 2.18E-23 |
| KCNN4 | 1.730918 | 17.94527 | 3.373994 | 1.53E-24 | 1.14E-23 |
| CDH23 | 0.711677 | 0.340786 | -1.06236 | 4.98E-20 | 2.18E-19 |
| CREB3L4 | 3.3878 | 9.650786 | 1.510298 | 5.94E-26 | 5.22E-25 |
| ABCB1 | 1.908949 | 0.719294 | -1.40813 | 2.14E-14 | 6.19E-14 |
| BGLAP | 0.368806 | 0.825508 | 1.162418 | 9.55E-07 | 1.53E-06 |
| PYGM | 0.557058 | 0.271224 | -1.03835 | 5.34E-22 | 2.93E-21 |
| GATA6 | 11.99964 | 3.945991 | -1.60453 | 2.71E-28 | 3.80E-27 |
| NLN | 1.312235 | 3.860651 | 1.556818 | 2.60E-32 | 1.11E-30 |
| SLC1A4 | 3.075978 | 7.323749 | 1.251537 | 5.13E-22 | 2.84E-21 |
| MYO19 | 1.180132 | 4.128747 | 1.806756 | 5.47E-34 | 4.70E-32 |
| TUBB1 | 0.926712 | 0.170485 | -2.44248 | 4.34E-28 | 5.72E-27 |
| LMNB2 | 6.641813 | 15.18364 | 1.192869 | 1.43E-19 | 6.08E-19 |
| ABCG2 | 3.891806 | 1.047589 | -1.89337 | 5.70E-30 | 1.08E-28 |
| DTYMK | 5.18755 | 13.7544 | 1.406768 | 2.77E-27 | 3.11E-26 |
| VIM | 320.7725 | 155.0503 | -1.04881 | 7.64E-28 | 9.67E-27 |
| HBA1 | 3.639753 | 0.1978 | -4.20172 | 2.33E-31 | 6.66E-30 |
| PNPLA6 | 26.29036 | 9.907712 | -1.40791 | 2.67E-32 | 1.11E-30 |
| SPTBN2 | 1.065089 | 6.568049 | 2.624491 | 3.90E-31 | 1.07E-29 |
| LCN2 | 19.34002 | 133.7049 | 2.789391 | 1.34E-12 | 3.39E-12 |
| SLC30A2 | 0.041697 | 0.378635 | 3.182801 | 9.05E-06 | 1.35E-05 |
| CA4 | 18.53024 | 1.012545 | -4.19382 | 3.12E-34 | 3.10E-32 |
| AGTR1 | 2.258445 | 0.711429 | -1.66654 | 8.88E-28 | 1.10E-26 |
| RCAN1 | 17.65876 | 5.643035 | -1.64584 | 5.01E-27 | 5.21E-26 |
| IL17A | 0.00785 | 0.039486 | 2.330641 | 9.31E-05 | 0.000132 |
| SLC4A11 | 0.789202 | 3.209212 | 2.023752 | 1.68E-10 | 3.57E-10 |
| RBP4 | 18.74764 | 5.116024 | -1.87361 | 1.14E-26 | 1.11E-25 |
| GJA1 | 82.93235 | 39.40576 | -1.07353 | 2.52E-19 | 1.06E-18 |
| ADH1B | 65.31024 | 7.733745 | -3.07807 | 4.72E-34 | 4.35E-32 |
| ANGPTL4 | 7.903415 | 23.79498 | 1.590109 | 3.12E-08 | 5.57E-08 |
| LIPA | 71.36661 | 33.63382 | -1.08534 | 3.58E-14 | 1.01E-13 |
| EPAS1 | 325.1145 | 54.08709 | -2.58759 | 8.10E-35 | 1.30E-32 |
| BIK | 1.975254 | 8.529409 | 2.110408 | 5.42E-22 | 2.96E-21 |
| DHFR | 1.421009 | 2.971057 | 1.064061 | 4.49E-22 | 2.51E-21 |
| CYBB | 63.4362 | 28.48505 | -1.1551 | 1.09E-17 | 4.15E-17 |
| AGER | 1096.032 | 37.75775 | -4.85937 | 4.08E-36 | 3.63E-33 |
| LTF | 11.62666 | 31.51266 | 1.438495 | 0.028511 | 0.032839 |
| SLC9A3R1 | 15.01708 | 31.76864 | 1.080999 | 6.07E-14 | 1.69E-13 |
| LDHA | 51.09425 | 141.9697 | 1.47435 | 3.71E-30 | 7.35E-29 |
| L2HGDH | 0.828107 | 1.749713 | 1.079228 | 1.46E-23 | 9.68E-23 |
| PRDX4 | 23.96977 | 68.97368 | 1.52483 | 7.79E-27 | 7.79E-26 |
| ERBB2 | 16.55201 | 37.24483 | 1.170033 | 7.02E-16 | 2.29E-15 |
| NQO1 | 11.45179 | 99.72122 | 3.122328 | 2.77E-23 | 1.77E-22 |
| COL7A1 | 0.298128 | 2.820982 | 3.242195 | 7.11E-20 | 3.09E-19 |
| GPBAR1 | 1.315424 | 0.396923 | -1.7286 | 4.61E-29 | 7.43E-28 |
| HKDC1 | 1.655909 | 5.835805 | 1.817308 | 1.07E-11 | 2.52E-11 |
| CLCNKA | 0.124539 | 0.359519 | 1.529467 | 0.015436 | 0.018218 |
| EIF2AK1 | 21.39624 | 46.58345 | 1.12246 | 4.37E-33 | 2.56E-31 |
| ADA | 1.687493 | 3.429364 | 1.02306 | 3.69E-11 | 8.33E-11 |
| FA2H | 1.448988 | 8.882499 | 2.61592 | 4.79E-21 | 2.36E-20 |
| TGFBR2 | 116.1315 | 40.60868 | -1.5159 | 7.55E-34 | 6.09E-32 |
| FGF2 | 2.567315 | 0.721668 | -1.83085 | 1.29E-28 | 1.94E-27 |
| NUDT1 | 3.433647 | 7.415442 | 1.110791 | 6.44E-21 | 3.15E-20 |
| NR5A1 | 0.005868 | 0.074252 | 3.6615 | 4.14E-07 | 6.92E-07 |
| ABCC2 | 0.120475 | 3.662775 | 4.926136 | 6.28E-08 | 1.10E-07 |
| CCL2 | 59.71354 | 23.98548 | -1.3159 | 8.87E-07 | 1.43E-06 |
| GAL | 0.065681 | 2.796339 | 5.41192 | 9.03E-18 | 3.46E-17 |
| ACSM2A | 0.011821 | 0.003215 | -1.87855 | 1.32E-15 | 4.18E-15 |
| NIPSNAP1 | 12.04917 | 28.1045 | 1.221867 | 1.97E-29 | 3.48E-28 |
| G6PD | 18.98468 | 52.21509 | 1.459631 | 1.89E-05 | 2.75E-05 |
| LRAT | 0.180113 | 0.727991 | 2.015022 | 4.66E-06 | 7.09E-06 |
| ATP1B1 | 86.28873 | 227.4318 | 1.39819 | 8.89E-16 | 2.88E-15 |
| ABCB11 | 0.012969 | 0.040646 | 1.648035 | 0.003921 | 0.004844 |
| PRKG1 | 3.62938 | 1.597162 | -1.18421 | 5.37E-24 | 3.78E-23 |
| CDH1 | 35.97208 | 79.27525 | 1.139993 | 3.24E-21 | 1.63E-20 |

*ConMean, mean value of the gene expression in normal samples;* *TreatMean, mean value of the gene expression in tumor samples; LogFC, Log fold change; FDR, false discovery rate.*

**Supplementary Table S2.** 131 differentially expressed lncRNAs related to mitochondrial homeostasis between tumor and normal samples.

| lncRNA | conMean | treatMean | logFC | pValue | fdr |
| --- | --- | --- | --- | --- | --- |
| LINC00973 | 0.023189 | 1.88166 | 6.342399 | 8.63E-09 | 1.98E-08 |
| MINCR | 1.199955 | 2.514281 | 1.067166 | 4.50E-13 | 1.63E-12 |
| AC018755.4 | 3.570456 | 1.290601 | -1.46807 | 7.04E-23 | 6.46E-22 |
| AC020907.4 | 0.770592 | 1.896474 | 1.299281 | 6.29E-09 | 1.45E-08 |
| AC027117.1 | 1.846787 | 4.787578 | 1.374278 | 0.002809 | 0.003621 |
| SNHG17 | 2.416379 | 5.342101 | 1.144561 | 1.89E-18 | 1.13E-17 |
| AC037198.1 | 0.447696 | 2.68774 | 2.585803 | 0.012661 | 0.01502 |
| AC083809.1 | 0.162555 | 5.545109 | 5.092218 | 0.000204 | 0.000292 |
| PVT1 | 0.405438 | 3.039957 | 2.906497 | 6.30E-33 | 3.93E-31 |
| AC131009.3 | 0.606665 | 1.668128 | 1.459259 | 4.40E-18 | 2.45E-17 |
| AL033397.1 | 0.032943 | 1.798015 | 5.770277 | 1.77E-07 | 3.61E-07 |
| AC127024.5 | 1.123263 | 2.525997 | 1.169157 | 1.32E-12 | 4.54E-12 |
| AL355574.1 | 1.070068 | 2.18714 | 1.031344 | 1.12E-14 | 4.79E-14 |
| AL031058.1 | 0.412759 | 1.918358 | 2.216499 | 1.31E-18 | 8.19E-18 |
| AL928654.2 | 1.387799 | 3.118673 | 1.168134 | 1.25E-08 | 2.82E-08 |
| SNHG3 | 2.535963 | 8.299911 | 1.710562 | 1.74E-23 | 1.75E-22 |
| AC087741.2 | 0.798433 | 1.881228 | 1.236431 | 2.03E-11 | 5.75E-11 |
| Z98257.1 | 0.062636 | 4.65304 | 6.215027 | 3.63E-26 | 5.66E-25 |
| AC092718.4 | 2.301462 | 5.056894 | 1.135701 | 1.73E-16 | 8.84E-16 |
| AC005332.5 | 1.506549 | 3.063838 | 1.024093 | 1.36E-06 | 2.56E-06 |
| AL589765.4 | 0.803461 | 2.544754 | 1.663227 | 6.79E-22 | 5.58E-21 |
| SNHG15 | 3.919905 | 8.399749 | 1.099528 | 1.11E-14 | 4.79E-14 |
| LINC00174 | 0.887451 | 1.774966 | 1.000052 | 4.39E-09 | 1.05E-08 |
| HIF1A-AS3 | 0.301159 | 2.231401 | 2.889351 | 6.46E-08 | 1.37E-07 |
| AL590666.2 | 0.194644 | 2.845013 | 3.869528 | 3.28E-18 | 1.86E-17 |
| DPP10-AS1 | 0.163252 | 1.765552 | 3.434951 | 0.000221 | 0.000314 |
| AP001107.9 | 3.684177 | 1.689723 | -1.12456 | 3.31E-23 | 3.13E-22 |
| AC026369.3 | 8.662691 | 0.968304 | -3.16128 | 7.69E-32 | 3.43E-30 |
| AC012640.4 | 0.737176 | 2.20143 | 1.57836 | 3.52E-19 | 2.34E-18 |
| LINC00665 | 0.640092 | 3.452512 | 2.431295 | 1.65E-14 | 6.96E-14 |
| SNHG25 | 0.728902 | 2.360976 | 1.695586 | 1.53E-15 | 7.25E-15 |
| AC008610.1 | 0.705229 | 1.965669 | 1.478857 | 3.68E-15 | 1.66E-14 |
| AL121832.2 | 0.947054 | 2.357119 | 1.315507 | 1.50E-11 | 4.33E-11 |
| LINC00106 | 1.355905 | 3.055347 | 1.17208 | 5.90E-06 | 1.00E-05 |
| AC010719.1 | 0.210588 | 1.717152 | 3.027524 | 1.24E-21 | 9.93E-21 |
| DRAIC | 0.268578 | 3.886927 | 3.855216 | 0.000873 | 0.001175 |
| AC138696.2 | 0.76456 | 1.749827 | 1.194512 | 9.76E-12 | 2.87E-11 |
| AC093278.2 | 4.246586 | 1.279868 | -1.73031 | 3.16E-29 | 8.95E-28 |
| ASMTL-AS1 | 1.24381 | 3.207625 | 1.366739 | 1.77E-06 | 3.27E-06 |
| AC007991.2 | 0.860402 | 2.52043 | 1.550588 | 0.026416 | 0.030413 |
| NEAT1 | 22.5693 | 55.28255 | 1.292463 | 0.025056 | 0.028954 |
| USP30-AS1 | 4.067572 | 1.866158 | -1.1241 | 1.70E-18 | 1.04E-17 |
| LINC01614 | 0.232575 | 3.240205 | 3.80032 | 6.77E-25 | 7.54E-24 |
| ZFPM2-AS1 | 0.040538 | 1.918371 | 5.56448 | 2.61E-25 | 3.13E-24 |
| AL158166.1 | 0.834027 | 1.946128 | 1.222441 | 4.93E-05 | 7.57E-05 |
| MIAT | 0.584209 | 2.219302 | 1.925549 | 3.03E-07 | 5.99E-07 |
| AP003352.1 | 1.063289 | 2.301776 | 1.114213 | 3.98E-14 | 1.63E-13 |
| LINC01655 | 0.464632 | 2.08385 | 2.165092 | 1.77E-05 | 2.82E-05 |
| AL445524.1 | 1.115697 | 8.264333 | 2.888953 | 1.06E-30 | 3.66E-29 |
| AC015813.1 | 1.170668 | 2.663656 | 1.186076 | 2.77E-06 | 4.94E-06 |
| AC132872.3 | 1.107425 | 2.244286 | 1.019047 | 1.04E-07 | 2.20E-07 |
| AC026355.2 | 0.367022 | 1.632179 | 2.152862 | 9.02E-07 | 1.75E-06 |
| AC104695.4 | 0.64158 | 2.554163 | 1.99315 | 0.001424 | 0.001882 |
| AL391244.2 | 0.497566 | 1.763528 | 1.825505 | 1.54E-26 | 2.83E-25 |
| AC091729.3 | 1.085335 | 2.476062 | 1.189908 | 2.89E-16 | 1.45E-15 |
| MIR4435-2HG | 1.18399 | 2.720586 | 1.200261 | 1.81E-21 | 1.38E-20 |
| LHFPL3-AS2 | 11.04935 | 2.915915 | -1.92194 | 3.16E-27 | 6.57E-26 |
| AC009237.15 | 1.181654 | 2.416677 | 1.032217 | 0.000525 | 0.000716 |
| PCCA-DT | 1.828475 | 6.543565 | 1.839436 | 5.19E-26 | 7.36E-25 |
| LINC00467 | 1.535747 | 3.387114 | 1.141116 | 1.61E-21 | 1.26E-20 |
| AC092279.1 | 1.066294 | 2.306536 | 1.113123 | 0.000344 | 0.00048 |
| FENDRR | 9.04397 | 0.687331 | -3.71788 | 2.42E-33 | 1.89E-31 |
| AC026785.3 | 0.003848 | 1.869602 | 8.924452 | 8.28E-12 | 2.48E-11 |
| AC090559.1 | 4.425518 | 1.638905 | -1.43311 | 1.71E-25 | 2.13E-24 |
| LINC00265 | 0.808272 | 1.77205 | 1.132507 | 9.74E-13 | 3.45E-12 |
| AL355488.1 | 0.676099 | 1.761474 | 1.381477 | 5.38E-11 | 1.47E-10 |
| AC006042.1 | 1.388673 | 4.147777 | 1.578631 | 1.14E-13 | 4.34E-13 |
| SRGAP3-AS2 | 7.542065 | 1.778157 | -2.08458 | 1.79E-13 | 6.73E-13 |
| AL365181.3 | 0.182407 | 4.291234 | 4.556162 | 9.27E-20 | 6.57E-19 |
| SNHG12 | 2.297354 | 5.375152 | 1.226332 | 1.58E-15 | 7.38E-15 |
| LRRK2-DT | 12.29947 | 3.671594 | -1.74412 | 1.01E-25 | 1.32E-24 |
| TBX5-AS1 | 4.264477 | 1.372511 | -1.63555 | 9.19E-27 | 1.79E-25 |
| AC010542.6 | 1.612227 | 3.318337 | 1.041405 | 1.32E-07 | 2.72E-07 |
| LMNTD2-AS1 | 1.290027 | 2.804262 | 1.12022 | 0.002736 | 0.003542 |
| SMIM25 | 22.76153 | 4.551109 | -2.32231 | 8.09E-33 | 4.21E-31 |
| DLGAP1-AS2 | 0.761129 | 1.597097 | 1.069239 | 4.49E-06 | 7.69E-06 |
| AL357093.2 | 4.795892 | 1.571148 | -1.60998 | 6.99E-11 | 1.90E-10 |
| PCAT19 | 6.474672 | 1.324507 | -2.28935 | 1.96E-33 | 1.89E-31 |
| LUCAT1 | 0.189314 | 2.060883 | 3.444409 | 3.89E-16 | 1.92E-15 |
| PCAT6 | 0.782499 | 5.601402 | 2.839627 | 1.89E-30 | 5.90E-29 |
| CYTOR | 1.66404 | 4.455845 | 1.421008 | 2.15E-22 | 1.82E-21 |
| LINC00942 | 0.038499 | 3.959325 | 6.684294 | 1.31E-22 | 1.17E-21 |
| LINC02471 | 5.403977 | 1.283932 | -2.07345 | 2.07E-26 | 3.58E-25 |
| SFTA1P | 84.11367 | 25.22797 | -1.73732 | 6.47E-26 | 8.78E-25 |
| AL109615.3 | 0.251375 | 2.202404 | 3.131169 | 4.68E-18 | 2.56E-17 |
| LINC02474 | 0.060553 | 1.840802 | 4.925993 | 9.74E-14 | 3.75E-13 |
| ANKRD44-AS1 | 7.267675 | 2.039801 | -1.83307 | 7.54E-14 | 2.94E-13 |
| EMSLR | 0.629129 | 2.967236 | 2.237693 | 3.82E-15 | 1.70E-14 |
| LINC01770 | 1.770579 | 3.961835 | 1.161948 | 1.60E-08 | 3.57E-08 |
| ANKRD10-IT1 | 5.073061 | 10.83378 | 1.094608 | 1.08E-07 | 2.27E-07 |
| MELTF-AS1 | 0.557751 | 1.870102 | 1.745423 | 5.06E-19 | 3.29E-18 |
| AP002498.1 | 0.105865 | 2.013337 | 4.249295 | 3.15E-18 | 1.82E-17 |
| AL365181.2 | 0.03921 | 1.700314 | 5.438441 | 7.84E-19 | 4.99E-18 |
| AC008735.2 | 1.315635 | 2.824681 | 1.102329 | 1.20E-08 | 2.74E-08 |
| AC010442.1 | 2.687826 | 5.967864 | 1.150775 | 7.96E-25 | 8.56E-24 |
| GAS5 | 25.77775 | 61.13343 | 1.245835 | 6.94E-15 | 3.05E-14 |
| AC048341.2 | 0.671113 | 2.559917 | 1.931469 | 1.34E-16 | 6.99E-16 |
| AL391427.1 | 0.124606 | 2.061938 | 4.04855 | 2.18E-07 | 4.42E-07 |
| CR936218.1 | 0.726426 | 1.590466 | 1.130561 | 0.000269 | 0.000378 |
| AL390719.2 | 0.849785 | 3.218602 | 1.921264 | 1.15E-12 | 4.02E-12 |
| AC010186.3 | 0.958308 | 2.438842 | 1.347634 | 3.97E-12 | 1.24E-11 |
| AC074117.1 | 0.789477 | 1.845504 | 1.225045 | 6.29E-21 | 4.67E-20 |
| LINC01176 | 0.872648 | 1.955527 | 1.164086 | 1.87E-10 | 4.91E-10 |
| PPP1R14B-AS1 | 0.211925 | 2.629697 | 3.633274 | 7.77E-34 | 1.89E-31 |
| LINC00511 | 0.327304 | 3.022619 | 3.207097 | 6.88E-31 | 2.68E-29 |
| AC093110.1 | 10.10276 | 1.241613 | -3.02446 | 1.98E-33 | 1.89E-31 |
| AC124319.1 | 0.420858 | 1.712462 | 2.024667 | 1.15E-09 | 2.83E-09 |
| MEG3 | 0.60342 | 2.953183 | 2.291037 | 0.002549 | 0.003313 |
| LINC01836 | 3.785921 | 1.312096 | -1.52877 | 2.40E-20 | 1.74E-19 |
| EP300-AS1 | 4.069242 | 1.38217 | -1.55783 | 2.17E-27 | 5.21E-26 |
| TMEM147-AS1 | 0.731678 | 1.671558 | 1.191913 | 6.65E-14 | 2.63E-13 |
| AL133355.1 | 4.72784 | 2.055869 | -1.20143 | 3.63E-26 | 5.66E-25 |
| AL691482.4 | 0.862604 | 3.905161 | 2.178611 | 5.27E-14 | 2.11E-13 |
| VPS9D1-AS1 | 0.416277 | 3.157152 | 2.923008 | 2.87E-27 | 6.40E-26 |
| LINC02257 | 0.596101 | 2.955561 | 2.309803 | 1.51E-06 | 2.81E-06 |
| AL590560.3 | 0.905211 | 2.189529 | 1.274295 | 9.72E-05 | 0.000146 |
| AC099850.4 | 1.16935 | 6.587927 | 2.494117 | 2.11E-24 | 2.20E-23 |
| SNHG1 | 3.337075 | 11.07366 | 1.730477 | 1.79E-27 | 4.66E-26 |
| AL035461.3 | 0.808378 | 1.748639 | 1.113131 | 2.13E-10 | 5.50E-10 |
| LENG8-AS1 | 1.017791 | 2.163327 | 1.08781 | 3.98E-14 | 1.63E-13 |
| DCST1-AS1 | 0.539753 | 1.915858 | 1.827619 | 5.06E-26 | 7.36E-25 |
| SLC9A3-AS1 | 0.77261 | 2.867807 | 1.892136 | 1.17E-11 | 3.41E-11 |
| AC136475.3 | 3.437958 | 16.05971 | 2.223822 | 2.29E-07 | 4.61E-07 |
| PITPNA-AS1 | 2.499566 | 5.174361 | 1.049703 | 2.55E-15 | 1.17E-14 |
| AP006621.2 | 0.71317 | 1.738278 | 1.285341 | 3.80E-06 | 6.59E-06 |
| LINC00482 | 0.697942 | 1.793189 | 1.361348 | 5.07E-06 | 8.65E-06 |
| AC079467.1 | 5.721007 | 1.396739 | -2.03421 | 2.32E-23 | 2.26E-22 |
| MAFG-DT | 1.532044 | 3.796685 | 1.309282 | 2.52E-18 | 1.48E-17 |
| MHENCR | 2.369731 | 5.235036 | 1.143476 | 9.51E-11 | 2.56E-10 |
| HM13-IT1 | 0.775454 | 1.648331 | 1.087892 | 4.45E-11 | 1.23E-10 |
| SLC25A25-AS1 | 0.62379 | 1.624776 | 1.381108 | 1.70E-11 | 4.87E-11 |

*ConMean, mean value of the gene expression in normal samples;* *TreatMean, mean value of the gene expression in tumor samples; LogFC, Log fold change; FDR, false discovery rate.*

**Supplementary Table S3.** The significantly enriched pathways in the high-risk group of LUAD patients.

| NAME | SIZE | NES | NOM  p-value | FDR  q-value |
| --- | --- | --- | --- | --- |
| KEGG_HUNTINGTONS_DISEASE | 180 | 2.341 | 0.000 | 0.000 |
| KEGG_PARKINSONS_DISEASE | 128 | 2.168 | 0.000 | 0.001 |
| KEGG_PROTEIN_EXPORT | 24 | 2.089 | 0.002 | 0.002 |
| KEGG_OXIDATIVE_PHOSPHORYLATION | 131 | 2.178 | 0.002 | 0.002 |
| KEGG_CITRATE_CYCLE_TCA_CYCLE | 31 | 2.098 | 0.000 | 0.002 |
| KEGG_AMINOACYL_TRNA_BIOSYNTHESIS | 41 | 2.117 | 0.000 | 0.002 |
| KEGG_SPLICEOSOME | 127 | 2.102 | 0.000 | 0.002 |
| KEGG_PYRIMIDINE_METABOLISM | 97 | 2.122 | 0.000 | 0.002 |
| KEGG_HOMOLOGOUS_RECOMBINATION | 28 | 2.066 | 0.000 | 0.002 |
| KEGG_ALZHEIMERS_DISEASE | 165 | 2.202 | 0.000 | 0.002 |
| KEGG_BASE_EXCISION_REPAIR | 35 | 2.045 | 0.000 | 0.002 |
| KEGG_RNA_POLYMERASE | 28 | 2.033 | 0.000 | 0.002 |
| KEGG_N_GLYCAN_BIOSYNTHESIS | 46 | 2.039 | 0.000 | 0.002 |
| KEGG_CELL_CYCLE | 124 | 2.028 | 0.002 | 0.003 |
| KEGG_PROTEASOME | 46 | 2.015 | 0.000 | 0.003 |
| KEGG_RNA_DEGRADATION | 59 | 1.995 | 0.002 | 0.004 |
| KEGG_PENTOSE_PHOSPHATE_PATHWAY | 27 | 1.983 | 0.000 | 0.004 |
| KEGG_DNA_REPLICATION | 36 | 1.962 | 0.000 | 0.006 |
| KEGG_MISMATCH_REPAIR | 23 | 1.942 | 0.004 | 0.007 |
| KEGG_PORPHYRIN_AND_CHLOROPHYLL_METABOLISM | 41 | 1.916 | 0.002 | 0.009 |
| KEGG_NUCLEOTIDE_EXCISION_REPAIR | 44 | 1.918 | 0.006 | 0.009 |
| KEGG_ASCORBATE_AND_ALDARATE_METABOLISM | 25 | 1.894 | 0.008 | 0.010 |
| KEGG_PURINE_METABOLISM | 158 | 1.886 | 0.002 | 0.011 |
| KEGG_ARGININE_AND_PROLINE_METABOLISM | 54 | 1.895 | 0.006 | 0.011 |
| KEGG_GLUTATHIONE_METABOLISM | 49 | 1.887 | 0.000 | 0.011 |
| KEGG_PENTOSE_AND_GLUCURONATE_INTERCONVERSIONS | 28 | 1.873 | 0.010 | 0.012 |
| KEGG_GLYOXYLATE_AND_DICARBOXYLATE_METABOLISM | 16 | 1.869 | 0.006 | 0.012 |
| KEGG_BASAL_TRANSCRIPTION_FACTORS | 35 | 1.826 | 0.006 | 0.017 |
| KEGG_OOCYTE_MEIOSIS | 112 | 1.813 | 0.014 | 0.018 |
| KEGG_TERPENOID_BACKBONE_BIOSYNTHESIS | 15 | 1.785 | 0.010 | 0.022 |
| KEGG_ONE_CARBON_POOL_BY_FOLATE | 17 | 1.774 | 0.008 | 0.024 |
| KEGG_PYRUVATE_METABOLISM | 40 | 1.753 | 0.018 | 0.027 |
| KEGG_FRUCTOSE_AND_MANNOSE_METABOLISM | 33 | 1.748 | 0.010 | 0.027 |
| KEGG_CYSTEINE_AND_METHIONINE_METABOLISM | 34 | 1.731 | 0.010 | 0.029 |
| KEGG_RIBOSOME | 88 | 1.732 | 0.039 | 0.030 |
| KEGG_GLYCOSYLPHOSPHATIDYLINOSITOL_GPI_ANCHOR_BIOSYNTHESIS | 25 | 1.686 | 0.028 | 0.039 |
| KEGG_MATURITY_ONSET_DIABETES_OF_THE_YOUNG | 25 | 1.649 | 0.027 | 0.048 |

*LUAD, lung adenocarcinoma; NES, normalized enrichment score; NOM, nominal; FDR, false discovery rate.*
